# Video Recording Patients for Direct Care Purposes: Systematic Review and Narrative Synthesis of International Empirical Studies and UK Professional Guidance

**DOI:** 10.2196/46478

**Published:** 2023-08-16

**Authors:** Rachael Lear, Sophia Ellis, Tiffany Ollivierre-Harris, Susannah Long, Erik K Mayer

**Affiliations:** 1 Imperial Clinical Analytics, Research & Evaluation (iCARE) London United Kingdom; 2 National Institute for Health and Care Research North West London Patient Safety Research Collaborative Institute of Global Health Innovation Imperial College London - St Mary's Hospital Campus London United Kingdom; 3 Imperial College Healthcare NHS Trust London United Kingdom; 4 Hillingdon NHS Foundation Trust London United Kingdom; 5 Imperial Clinical Analytics, Research & Evaluation (iCARE) Digital Collaboration Space London United Kingdom; 6 National Institute for Health and Care Research North West London Patient Safety Research Collaborative Institute of Global Health Innovation Imperial College London London United Kingdom

**Keywords:** video recording, audio-visual media, digital health, electronic health records, mobile phone

## Abstract

**Background:**

Video recordings of patients may offer advantages to supplement patient assessment and clinical decision-making. However, little is known about the practice of video recording patients for direct care purposes.

**Objective:**

We aimed to synthesize empirical studies published internationally to explore the extent to which video recording patients is acceptable and effective in supporting direct care and, for the United Kingdom, to summarize the relevant guidance of professional and regulatory bodies.

**Methods:**

Five electronic databases (MEDLINE, Embase, APA PsycINFO, CENTRAL, and HMIC) were searched from 2012 to 2022. Eligible studies evaluated an intervention involving video recording of adult patients (≥18 years) to support diagnosis, care, or treatment. All study designs and countries of publication were included. Websites of UK professional and regulatory bodies were searched to identify relevant guidance. The acceptability of video recording patients was evaluated using study recruitment and retention rates and a framework synthesis of patients’ and clinical staff’s perspectives based on the Theoretical Framework of Acceptability by Sekhon. Clinically relevant measures of impact were extracted and tabulated according to the study design. The framework approach was used to synthesize the reported ethico-legal considerations, and recommendations of professional and regulatory bodies were extracted and tabulated.

**Results:**

Of the 14,221 abstracts screened, 27 studies met the inclusion criteria. Overall, 13 guidance documents were retrieved, of which 7 were retained for review. The views of patients and clinical staff (16 studies) were predominantly positive, although concerns were expressed about privacy, technical considerations, and integrating video recording into clinical workflows; some patients were anxious about their physical appearance. The mean recruitment rate was 68.2% (SD 22.5%; range 34.2%-100%; 12 studies), and the mean retention rate was 73.3% (SD 28.6%; range 16.7%-100%; 17 studies). Regarding effectiveness (10 studies), patients and clinical staff considered video recordings to be valuable in supporting assessment, care, and treatment; in promoting patient engagement; and in enhancing communication and recall of information. Observational studies (n=5) favored video recording, but randomized controlled trials (n=5) did not demonstrate that video recording was superior to the controls. UK guidelines are consistent in their recommendations around consent, privacy, and storage of recordings but lack detailed guidance on how to operationalize these recommendations in clinical practice.

**Conclusions:**

Video recording patients for direct care purposes appears to be acceptable, despite concerns about privacy, technical considerations, and how to incorporate recording into clinical workflows. Methodological quality prevents firm conclusions from being drawn; therefore, pragmatic trials (particularly in older adult care and the movement disorders field) should evaluate the impact of video recording on diagnosis, treatment monitoring, patient-clinician communication, and patient safety. Professional and regulatory documents should signpost to practical guidance on the implementation of video recording in routine practice.

**Trial Registration:**

PROSPERO CRD42022331825: https://www.crd.york.ac.uk/prospero/display_record.php?RecordID=331825

## Introduction

### Background

Video-based documentation is a growing practice in health care, but both clinicians and patients have concerns about associated ethico-legal issues [1,2]. Video recordings capturing specific aspects of a patient’s condition may offer advantages that could support patient assessment and monitoring, support clinical decision-making, and lead to better clinical outcomes. However, little is known about the application, acceptability, and impact of video recordings for direct care purposes.

### Video Recording Applications in Health Care

Video recording has demonstrated value across research, education, and performance assessment, as well as in clinical audit and quality improvement [1,2]. Researchers value the richness of video data that include sound, environmental context, and body language, which facilitate the objective and accurate documentation of behavior [3,4]. Video-based feedback assists clinicians in recognizing visual and auditory clues during clinical consultations that cannot be derived from text-based learning, and video-based curricula have led to an accelerated learning curve for surgical trainees [2]. Furthermore, video-based surgical case reviews have informed quality improvement initiatives through the provision of increased detail and nuance beyond what exists in operative notes alone [2,5-7].

### Ineffective Communication

Certain aspects of a patient’s condition (such as functional ability, cognition, and behavioral symptoms) can be challenging to convey to others involved in a person’s care, and recognizing changes in a patient’s condition over time can be difficult, particularly when a patient receives care from multiple different clinical staff [8]. Moreover, information captured about a patient away from the clinical setting can be useful to inform diagnosis, care, and treatment (eg, symptoms occurring in the home). As ineffective communication of pertinent patient information is known to negatively affect care continuity and is linked to medical errors and adverse events [9,10], new consideration should be given to how best to capture and communicate these data [11].

### Video Recording Advantages

One of the proposed solutions is video recording. Video recordings offer a detailed and objective record that can be reviewed repeatedly as a longitudinal record by multiple clinical staff members [8]. Video recordings provide a record that has not been condensed into words and thus preserves the original situation without the added lens of a patient, family member, or intermediary health professional [7]. However, privacy concerns and issues with storage in the electronic patient record have limited the use of video recording for direct care purposes [12,13]. Early studies suggested that patients can feel censored or self-conscious in front of a camera, and video recording can alter the dynamic between the patient and clinician in negative ways [13,14]. However, over the last decade, the proliferation of smartphones has resulted in photographs and videos being captured in everyday life and being used for agile communication. Technological advances are supporting lawful collection, handling, and cloud-based storage of visual data to address storage limitations and protect people’s privacy in line with data protection legislation [15-18].

At a time when video technology is ubiquitous in everyday life, little is known about how video recordings can support the safety and quality of individual patient health care. The aim of this review was to synthesize the international empirical literature on video recording patients for direct care purposes to evaluate acceptability, effectiveness, and ethico-legal considerations and to summarize the relevant guidance of professional and regulatory bodies in the United Kingdom.

## Methods

### Protocol and Registration

The review protocol was specified in advance and registered in the PROSPERO database (CRD42022331825).

### Search Strategy and Eligibility Criteria

Two separate search strategies were used: (1) empirical studies (international) and (2) professional and regulatory guidance (United Kingdom only).

#### Empirical Studies

Empirical studies were identified by searching electronic databases (MEDLINE, Embase, APA PsycINFO, CENTRAL, and HMIC) and by scanning the reference lists of relevant articles. The literature search was based on two broad concepts: (1) video recording and (2) patients (Multimedia Appendix 1). Studies were eligible for inclusion if they evaluated an intervention involving video recording adult patients, service users, or care home residents (aged ≥18 years) for the purposes of providing direct care in a health or care setting (Table 1). All study designs and countries of publication were considered. Direct care was defined as activities that directly contribute to the diagnosis, care, and treatment of an individual [19]. Articles were excluded if the video recordings were taken solely for research data collection or for medical education or training purposes.

**Table 1:** Eligibility criteria.

Inclusion criteria			Exclusion criteria
**Empirical studies**			
	**Population**		
		Adult patients, service users, or care home residents Aged ≥18 years Any diagnosis or medical condition With or without capacity to consent to study participations and being video recorded	Children or young people aged ≤17 years
	**Intervention**		
		Video recording patients for direct care purposes^a^ Patients are identifiable in the video recordings	Video recording patients for education or training purposes Video recording patients for research purposes Video surveillance for security or audit Medical imaging (eg, ultrasound) Surgical site video recordings Telemedicine and use of video without a recording being taken Audio recordings only Photographs
	**Comparison**		
		Any or none	N/A^b^
	**Outcomes or end points**		
		Acceptability Effectiveness or efficacy Ethico-legal considerations	Any other outcomes or end points
	**Study design**		
		Primary research or review papers examining one or more of the outcomes of interest (any study design or any country of publication) Published since 2012	Editorials, commentaries, and expert opinion Published before 2012
**Professional and regulatory guidance**			
	**Population**		
		—^c^	—
	**Intervention**		
		—	—
	**Outcomes or end points**		
		Key recommendations	Any other outcomes or end points
	**Document types**		
		Policies, guidelines, and recommendations of national governmental, professional, and regulatory bodies (United Kingdom only) Published since 2012	Policies, guidelines, and recommendations of local organizations Non-UK policies, guidelines, and recommendations Published before 2012

^a^Video recording interventions that directly contribute to the diagnosis, care, or treatment of an individual.

^b^N/A: not applicable.

^c^As per criteria for empirical studies.

#### Professional and Regulatory Guidance

To identify relevant recommendations of professional and regulatory bodies, we searched the following websites and archives: National Institute for Health and Care Excellence, Social Care Institute for Excellence, National Health Service Knowledge Library Hub, General Medical Council, Nursing & Midwifery Council, Health & Care Professions Council, Care Quality Commission, Parliamentary & Health Service Ombudsman, and Government publications. Similar to the search strategy for empirical studies, the search was underpinned by two concepts: (1) video recording and (2) patients. As we specified our review protocol in advance and anticipated that it would not be feasible to cover all countries, we opted to include professional and regulatory guidance documents issued in the United Kingdom where the authors are based.

For both search strategies, the last search was conducted on December 13, 2022, and we excluded papers that were older than 10 years or not published in English [20].

### Study Selection and Data Extraction

#### Overview

Two reviewers (RL and SE, both with clinical, research, and academic backgrounds) independently screened all the records. The reviewers were blinded to each other’s screening decisions, and disagreements were resolved through discussion and consensus. Screening decisions were recorded using Covidence, a web-based systematic review management system.

#### Empirical Studies

Both reviewers independently extracted the following data from the included studies: authors and year of publication; study design and type of publication (eg, conference abstract or full journal publication); country of publication and clinical setting; focus of the paper; details regarding the video recording intervention (intervention goal, equipment used, procedure, etc); sample size and participant characteristics (age, sex, ethnicity, and medical problems or diagnoses); main findings relating to the outcomes of interest (acceptability, effectiveness, and ethico-legal considerations); and study limitations. Acceptability was defined as the extent to which people delivering or receiving health care interventions consider it appropriate based on anticipated or experienced cognitive and emotional responses to the intervention [21]. Effectiveness was defined as the ability of an intervention to have a meaningful effect on a patient in normal clinical conditions [22].

#### Professional and Regulatory Guidance

Key recommendations for video recording patients for direct care purposes were extracted independently by the reviewers.

### Quality Appraisal of Empirical Studies

Owing to the inclusion of various types of evidence encompassing a variety of study designs and qualitative, quantitative, and mixed methods approaches, quality appraisal was undertaken using the Quality Assessment with Diverse Studies (QuADS) appraisal tool [23]. The use of a single tool permits the evaluation of methodological quality, evidence quality, and quality of reporting across a body of diverse evidence, and the QuADS tool considers the extent to which there is transparency and congruency in the research and its reporting and the implications for evidence quality [23].

We used the Joanna Briggs Institute Critical Appraisal Checklist for Case Reports [24]. For conference abstracts, we devised a bespoke appraisal tool based on the STROBE (Strengthening the Reporting of Observational Studies in Epidemiology) checklist of items to be included when reporting observational studies in conference abstracts [25]. Study quality was assessed independently by 2 reviewers.

### Synthesis

The study’s findings were synthesized using a narrative approach. To evaluate acceptability, recruitment and retention rates were extracted or calculated (reported data permitting), and the reported views and experiences of patients and clinical staff were analyzed thematically using a framework-based synthesis [20]. The Theoretical Framework of Acceptability (TFA) of health care interventions (Textbox 1) was applied as a preliminary coding framework, and an inductive approach was used to derive new themes from the data [21]. The framework approach was also applied to synthesize reported ethico-legal considerations. To evaluate effectiveness, we selected studies reporting any clinically relevant, objective measure of impact of the video recording intervention, considering the results of observational studies and randomized controlled trials (RCTs) separately, because although RCTs are usually considered to be the gold standard design for decisions about the effect of interventions, they are arguably less applicable to real-world clinical practice than observational studies, and this review was concerned with direct care delivery [22]. Key recommendations for professional and regulatory bodies are summarized and tabulated.

**Definitions of the component constructs of the Theoretical Framework of Acceptability.**

**Affective attitude**

- How an individual feels about the intervention

**Burden**e

- The amount of effort required to participate in the intervention

**Ethicality**

- The extent to which the intervention has a good fit with an individual’s value system

**Intervention coherence**

- The extent to which the participant understands the intervention and how it works

**Opportunity costs**

- The extent to which benefits, profits, or values must be given up to engage in the intervention

**Perceived effectiveness**

- The extent to which the intervention is perceived to achieve its purpose

**Self-efficacy**

- The participant’s confidence that they can perform the behaviors required to participate in the intervention

### Reporting

We followed the reporting recommendations outlined in the PRISMA (Preferred Reporting Items for Systematic Reviews and Meta-Analyses) statement [26] (Multimedia Appendix 2).

## Results

### Overview

**Figure 1:**
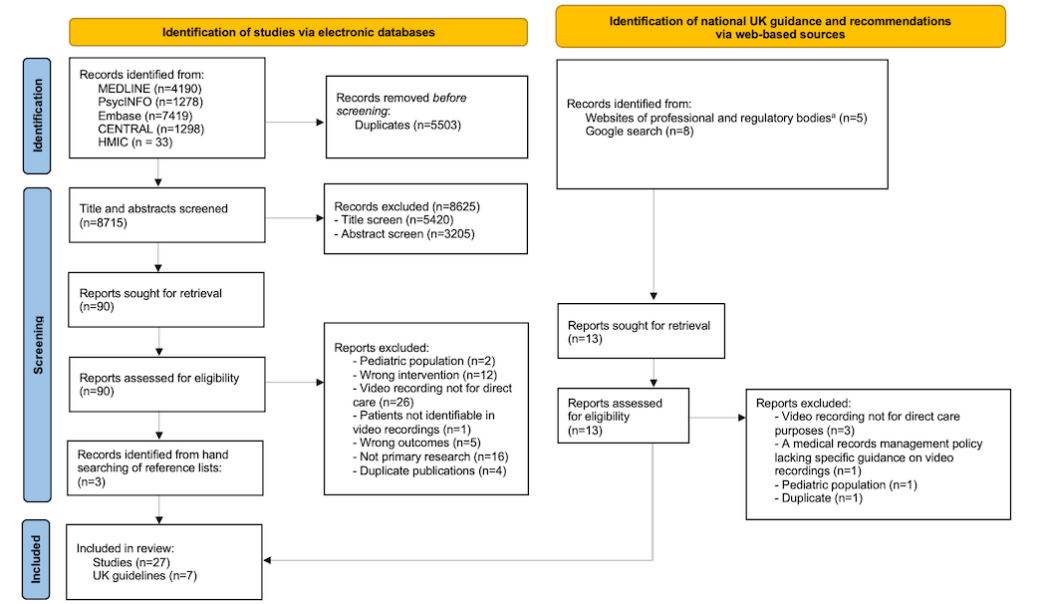
PRISMA (Preferred Reporting Items for Systematic Reviews and Meta-Analyses) flow diagram. aWebsites of public & regulatory bodies searched: Care Quality Commission; General Medical Council; Health & Care Professions Council; National Health Service Knowledge Library Hub; Nursing & Midwifery Council; Parliamentary & Health Service Ombudsman; Social Care Institute for Excellence; UK Government.

The searches of the MEDLINE, Embase, APA PsycINFO, CENTRAL, and HMIC databases yielded 14,218 study citations. Following the removal of duplicates, the titles with the addition or removal of abstracts of 8715 citations were screened. Of these, 5420 citations that clearly did not meet the inclusion criteria were discarded during the title screen, and a further 3205 were excluded on reviewing the abstracts. The full reports of the remaining 90 studies were retrieved and examined in detail. In total, 24 studies meeting the inclusion criteria were retained for review; 3 additional studies were identified through a hand search of reference lists (Figure 1). A total of 13 guidance documents were identified via the websites of public and regulatory bodies and Google search, of which 7 met the inclusion criteria and were retained for the review.

### Study Characteristics

#### Overview

Of the 27 studies, there were 5 RCTs [27-31], 17 observational studies [32-48], and 5 case reports [49-53] (Multimedia Appendix 3 [27-53]). In total, 22 studies were full reports in academic journals [27,28,30-34,36-39,41-45,47,48,50-53] and 4 were conference abstracts [29,35,40,46], and there was 1 doctoral thesis [49]. All the studies were published in English between 2012 and 2022. A total of 17 studies were conducted in the United States [29,30,32,34-39,41,42,46,47,49-51,53] (including 1 binational study: United States and Mexico [34]), 3 in the United Kingdom [27,28,45], 2 in the Netherlands [33,48], 2 in India [40,44], 1 in Spain [43], 1 in Portugal [52], and 1 in France [31].

#### Participants

The studies involved 1551 patient participants. Patients were care home residents and older people with dementia [32,38,39,42]; adults with seizures [41,43,44,50]; hospital inpatients with stroke [28,29], cancer [35,36], or mental health disorders [27,31,33]; surgical outpatients [30,47,53]; and patients with Parkinson disease [40,48], musculoskeletal disorders [49], tuberculosis [34], and dysphagia [6,9,12-14,16-18,21,25,45]. Clinical staff member’s experiences and perceptions regarding the video recording intervention were reported in 16 studies [28,32,34,37-41,43-45,49-53].

#### Video Recording Interventions

In 11 studies, patient video recordings were used to support assessment and diagnosis, including the evaluation of seizures [41,43,44,50,52], gait or movement patterns [40,48,49,51,53], and dysphagia [45]. In 7 studies, video recordings were used for treatment purposes: patients with stroke or musculoskeletal disorders were video recorded during physiotherapy sessions, who then viewed the recordings to support their rehabilitation [28,29,49]; in 3 mental health studies, patients watched video recordings of themselves to enhance insight into obsessive compulsive or psychotic behaviors (“video self-confrontation”) [27,31,33]; and in a study of medication adherence, patients submitted home videos of themselves ingesting their tablets to their clinical team [34]. In total, 9 studies evaluated the application of video recordings for patient care [32,35-39,42,46,47]: in 2 studies, care home staff members reviewed video monitoring footage to support postfall care [32,42] and, in 1 study, in-home video monitoring enabled clinical staff members to support informal caregivers of people with dementia [39]. In other studies, video recordings were used to communicate patients’ care preferences [35-38], enhance patients’ recall of clinical information [30], and support patient-provider communication [46,47]. Most video recording interventions use mobile devices (ie, mobile phones or tablets) for video capture; some used specialized applications or software [30,32,34,39,42,48,49].

### Quality Assessment

In total, 17 empirical studies were reported as full-text articles and were therefore suitable for methodological assessment using the QuADS appraisal tool [27,28,30-34,37-39,41-45,47,48]. Methodological and reporting quality was generally poor: only 4 studies achieved a maximum score of 3 in ≥10 of the 13 QuADS criteria [28,31,33,34] (Table S1 in Multimedia Appendix 4 [28-53]). Overall, QuADS scores tended to be higher in RCTs (mean total score 32.8, range 27-39; 4 RCTs) than in observational studies (mean total score 27.8, range 17-36; 13 observational studies). The quality of the 5 conference abstracts was mixed (Table S2 in Multimedia Appendix 4). The Joanna Briggs Institute Critical Appraisal Checklist indicated satisfactory quality of the 5 case reports (Table S3 in Multimedia Appendix 4).

### Synthesis

#### Acceptability

##### Recruitment and Retention Rates

The mean recruitment rate was 68.2% (range 34.2%-100%; figures based on 12 studies that reported recruitment data; Multimedia Appendix 5 [27-29,32-35,40-48]). The mean retention rate was 73.3% (range 16.7%-100%; figures based on 17 studies that reported retention data). Reasons for nonparticipation or withdrawal related to both the video recording intervention itself (eg, privacy concerns and feeling self-conscious about being recorded) and to other factors mostly related to the patient’s condition (eg, low frequency or short duration of seizures precluded video capture; Multimedia Appendix 5).

##### Framework Synthesis

**Figure 2:**
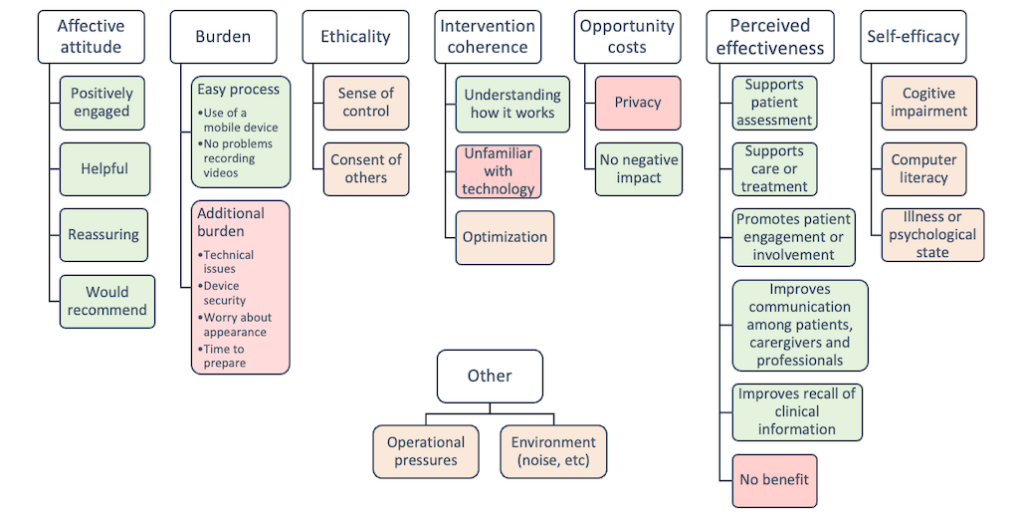
Acceptability of video recording patients for direct care: Theoretical Framework of Acceptability constructs with linked themes and “other” emergent themes.

The TFA was systematically applied to 16 studies that reported the perspectives and experiences of the patients and clinical staff. Framework synthesis generated 23 themes linked to the 7 TFA constructs; two additional themes not covered by the TFA also emerged: (1) operational pressures and (2) environment (noise, etc; Figure 2). Additional verbatim quotes for each theme are provided in Multimedia Appendix 6 [21,28-30,32-39,41,45-48].

##### Affective Attitude

Stakeholder reports coded to this construct were universally positive. Many patients and service users were positively engaged in being video recorded and were eager to share their video recordings with people involved in their care [28,30,45]. Patients’ families and clinical staff members described the video recordings as “helpful” [35-37,47] and “reassuring” [28,46], and most said they would recommend video recording to other patients [35-37,47].

##### Burden

Stakeholder reports were mixed for this construct. Some patients and clinical staff reported that video recording processes were “easy,” particularly when mobile devices (smartphones or tablets) were used [32,34,38,39,45]:

The mobile device app provided to review videos proved to be accessible and easy to use to facility staff.

Results extract [32]

Others described an additional burden: for example, some clinical staff members were concerned about losing the mobile video recording devices in the clinical setting; others described technical problems in operationalizing the video recording intervention [28,37,39,47]. Some patients “wished their appearance in the video was better” [31,37,38] while others said they needed time to prepare for being recorded [37,38].

##### Ethicality

Patients’ reports coded to this construct were from 1 study, which explored perspectives on home-based video recording for movement analysis in Parkinson disease [1,2,48]. For most patients, it was important to have a “sense of control over the camera” (participant-patient 16 [48]; eg, the opportunity to delete recordings). Some patients pointed out that it would be important to obtain consent from other persons appearing in the videos (eg, family members) [48]:

Other people should be kept out of the video as much as possible... but this is probably not possible, so my partner needs to consent as well.

Participant-patient 7

##### Intervention Coherence

It was clear from some patients’ reports that they clearly understood how the video recording intervention worked [28,33,45]; for example, patients with obsessive compulsive disorder believed that by observing their own compulsions, they gained more insight into their illness and became more motivated to change [33]. However, video recording technology is unfamiliar to some service users with learning disabilities [45]:

Some service-users showed curiosity about the iPad and appeared not to recognise or understand it. One person asked if it was a mirror and looked to brush her hair, another asked if it could continue to see him in another room.

Results extract

In 1 study [28], therapists suggested that a video-guided exercise intervention for patients with stroke could be optimized by “using older videos [of patients] to demonstrate progress and goal attainment.”

##### Opportunity Costs

In 1 study [48] in which the intervention involved home-based video recording for movement analysis, some participants were concerned about the impact on their personal privacy:

I don’t want a camera in every room at the same time, I need to have some privacy somewhere.

Patient 8; patient with Parkinson disease

However, in another study [47] where clinical medical encounters were video recorded, “no patient made a comment that the video was intrusive.” A further study [30] involving video recording clinical encounters reported that the incorporation of video recordings into the clinical workflow “did not add any significant time to the visit.”

##### Perceived Effectiveness

Video recordings were perceived to be valuable in supporting patient assessment, including assessing condition severity and monitoring changes [32,33,45]. Specifically, video recordings of patients were considered to have certain advantages, including the “true visual representation” (study participant—speech and language therapist) and “depth of information not usually achieved by written transcription of assessment alone” (Results extract) [45]. Patients and clinical staff considered video recordings to be valuable for remote assessments [34,39,45] and removed the need to coordinate the availability of specialist clinical staff with the timing of specific events (eg, mealtimes and dysphagia assessment [45]). Patients and clinical staff members perceived video recordings to be effective in supporting care or treatment in different ways; for example, to inform fall prevention measures [32], to guide patients undertaking physical therapy after stroke [28,29], and to improve patient insight into obsessive compulsive disorder [33]. When an intervention involved patients viewing their video recordings, the videos were considered valuable in promoting patient engagement in their own health care [28,33,34,45]. Video recordings were perceived to improve communication; for example, some patients and their families felt that video recordings better conveyed problems, symptoms, care preferences, or clinical information [37-39,47,48]:

When you visit a neurologist you really need to describe the problem the right way and you never know exactly how often symptoms appear.

Patient 10 [48]

Clinical staff members reported that patient videos supported multidisciplinary communication as well as referrals for advice or a second opinion [45]. Video recordings were also perceived to improve the recall of information about a patient’s previous condition or clinical encounters [28,45,47]. In 2 studies, some patients believed that the video recordings had no benefit [38,47].

##### Self-Efficacy

Four studies reported factors affecting patients’ ability to engage with or participate in video recording interventions; these were as follows: cognitive impairment—“Cognitively impaired patients were less likely to engage with the tablet” (results extract) [28]; computer literacy—“respondents...indicated it was difficult for them to figure out how to get to the video” (results extract) [47]; and illness or psychological state [29,30].

##### Other

In 1 study [32], operational pressures meant that the video recording intervention (video monitoring and review of falls) was not used in the first few weeks of implementation, “because of numerous other challenges faced with operating a memory care facility and the little obvious value granted to the video so far” (results extract). In 2 studies [27,33], patients commented that there was too much noise or insufficient space in clinical areas and that these environmental challenges affected how well they could participate in the video recording intervention.

#### Effectiveness

Effectiveness was quantitatively measured in 10 studies. Of these, 5 were observational and examined the use of home videos as adjunct tools in epilepsy diagnosis [41,43,44] or the impact of continuous video monitoring on falls incidence and postfall care provision [32,42]. All 5 observational studies reported results favoring the impact of the video recording intervention on specific process measures (Table 2). However, among the 5 RCTs (which examined the impact of the intervention on patient outcomes), no study demonstrated that the video recording intervention was superior to controls (Table 3). Video self-confrontation did not improve patients’ insight into their psychotic behaviors [27,31], and providing rhinology patients with access to video recordings of their clinical visits did not improve their satisfaction with care or recall of procedural risks [30]. Showing stroke patients video recordings of the gait during inpatient rehabilitation may have a positive effect on functional outcomes, but the sample size for this study was small (N=23) [29]. A randomized feasibility trial reported that functional outcomes for video-guided exercise appeared to be similar to treatment-as-usual; however, the results of this pilot study were only indicative of possible effects [28].

**Table 2:** Objective measures of effectiveness: results of observational studies.

Study, year		Intervention goal	Comparator	Measure of effectiveness	Main results	Interpretation of results^a^
**Diagnosis**						
	Amin et al [41], 2021	Use home videos of seizures as an adjunct tool in epilepsy diagnosis	Video EEG^b^ (gold standard)	Accuracy of home videos in distinguishing between epileptic and nonepileptic events	Of 17 events that were confirmed as epileptic by video EEG monitoring, the home video correctly predicted 13 (PPVc=76%). Of 23 patients whose final diagnosis was nonepileptic event, the home video correctly predicted the diagnosis in 21 (PPV=91.	Home video interpretation is a useful adjunctive tool in the diagnosis of seizures.
	Dash et al [44], 2016	Use home videos of seizures as an adjunct tool for classification of epilepsy type	Video EEG (gold standard)	Yield of semiological features and classification of epilepsy type	Mean number of semiology features inferred from home videos was 3.3 (SD 2.2) and from the caregiver history was 2.1 (SD 1.1; *P*<.01). From video EEG (gold standard)=4.9 (SD 1.5). Interobserver agreement (Cohen κ): caregiver history vs video EEG=0.75; home videos vs video EEG=0.92. Using caregiver history, 50.3% of patients correctly classified as having focal epilepsy. Using home videos, 74.5% of patients correctly classified as having focal epilepsy.	Home videos are a complementary tool in epilepsy classification.
	Ojeda et al [43], 2016	Use home videos of seizures to improve diagnostic accuracy	Previous diagnosis obtained from clinical records	Proportion of patients with confirmed or revised diagnosis	82% (18/22) of patients were confirmed in their previous diagnosis after a review of home videos. The diagnosis of 14% (3/22) of patients was revised; home videos indicated nonepileptic seizures. One patient was considered with no defined diagnosis (no agreement in the nature of the event captured on home video among experts).	Home videos of seizures may be of diagnostic value in epilepsy management.
**Care**						
	Bayen et al [32], 2017	Use continuous video monitoring and review of falls video footage to improve quality of care for residents in memory care facilities	Pre-post intervention design	Comparison of falls rate between baseline and intervention periods Changes to care practices	Reduction in falls rate of 18 falls/mo to 2 falls/mo. Implementation of secondary prevention strategies in high-risk multifaller individuals; updated facility care policies for a safer environment.	Video monitoring of residents and review of falls footage by facility staff has a positive impact on the quality of care in memory care facilities.
	Bayen et al [42], 2021	Use AI^d^-enabled continuous video monitoring with real-time falls notifications to improve early postfall care provision.	Pre-post intervention design	Comparison of mean TUAe and mean TOGf between baseline and intervention periods.	TUA: reduction of 28.3 (95% CI 19.6-37.1) min TOG: reduction of 29.6 (95% CI 20.3-38.9) min Proportion of fallers with TOG >60 min fell from 31% (8/26; baseline) to 0% (0 fall events during the intervention period).	AI-enabled continuous video monitoring with real-time falls notifications improves postfall care in memory care facilities. The substantial reduction in time on the ground may decrease secondary complications related to postfall immobilization.

^a^Results favor the video recording intervention.

^b^EEG: electroencephalogram.

^c^PPV: positive predictive value.

^d^AI: artificial intelligence.

^e^TUA: time until staff assistance.

^f^TOG: time on the ground.

**Table 3:** Objective measures of effectiveness: results of randomized controlled trials.

Study, year		Intervention goal	Control group	Measure of effectiveness	Main results	Interpretation of results
**Care**						
	Sharma et al [30], 2018	Provide rhinology patients with access to video recordings of their clinical encounter to improve recall and satisfaction.	Patients denied access to video recordings of their clinical encounter.	Patient recall of procedural risks Patient satisfaction	Correct patient recall of procedural risks: 66% (19/29) in the intervention group; 63% (7/11) in the control group (*P*>.05). Average patient satisfaction score: 4.57 in the intervention group; 4.57 in the control group.	Providing rhinology patients with access to video recordings of their clinical encounter does not improve patient recall or satisfaction^a^.
**Treatment**						
	David et al [27], 2012	Show patients video recordings of their psychotic behaviors to improve insight into illness and treatment.	Show patients videos of an actor presenting with psychotic behaviors.	Differences in insight scores between “self” video and “actor” video, as measured by 2 instruments: SAE-I^b^ and ITAQ^c^.	Insight measures increased during treatment in both video groups. SAE-I: nonsignificant difference between the 2 video groups, favoring the “self” video (coefficient 1.192, 95% CI −0.036 to 2.42; *t*=1.97; *P*=.052). ITAQ: nonsignificant difference between the 2 video groups, favoring the “actor” video (coefficient −1.286, 95% CI −2.64 to 0.068; *t*=1.92; *P*=.06).	The beneficial effect of video confrontation is not specific to viewing video recordings of the self but rather any person with psychotic symptoms^a^.
	Jayabalan et al [29], 2014	Show stroke patients video recordings of their gait to improve inpatient rehabilitation outcomes	Treatment-as-usual (no video)	Differences between groups for the following: Change in the TUG^d^ test from admission to discharge Change in 10-m walk test from admission to discharge. Length of stay	“The degree of change in TUG from admission to discharge significantly improved in the video recording group compared to controls (*P*<.05)” (detailed results not reported). Results not reported for the 10-m walk test. “There was also a non-significant decrease in length of stay in the recording group (16.3 days) compared to the control group (17.2 days).”	Showing stroke patients video recordings of their gait during inpatient rehabilitation may have a positive effect on functional outcomes^e^.
	Kenny et al [28], 2020	Use video-guided exercise to improve upper limb outcomes after stroke.	Treatment-as-usual (no video)	Difference between groups for the mean change in Motor Status Score	Mean change in Motor Status Score for the control group was 18.8 (95% CI 8.9-28.7) and for the intervention group was 18.9 (95% CI 8.1-29.8).	Functional outcomes for video-guided exercise appear to be similar to treatment-as-usual. The authors emphasize that the results of this pilot study are only indicative estimates of possible effects^e^.
	Schandrin et al [31], 2022	Show patients video recordings of their psychotic behaviors to improve insight into illness	Treatment-as-usual (no video)	Change in SUMD^f^ from baseline to 48 h, 1 mo, and 4 mo	No difference between groups in change in the global SUMD score between inclusion and the visit at 48 h (1.98+0.02 no video vs 1.88-0.01 self-video group, *P*=.98) or at 1 and 4 mo.	Video self-confrontation did not change the levels of insight^a^.

^a^Results that are unclear or indicate that the video recording intervention is not superior to controls.

^b^SAE-I: Schedule for Assessment of Insight—expanded version.

^c^ITAQ: Insight & Treatment Attitudes Questionnaire.

^d^TUG: Timed Up & Go.

^e^Results demonstrate that the video recording intervention is not effective.

^f^SUMD: Scale to assess Unawareness of Mental Disorder.

#### Ethico-Legal Considerations

**Figure 3:**
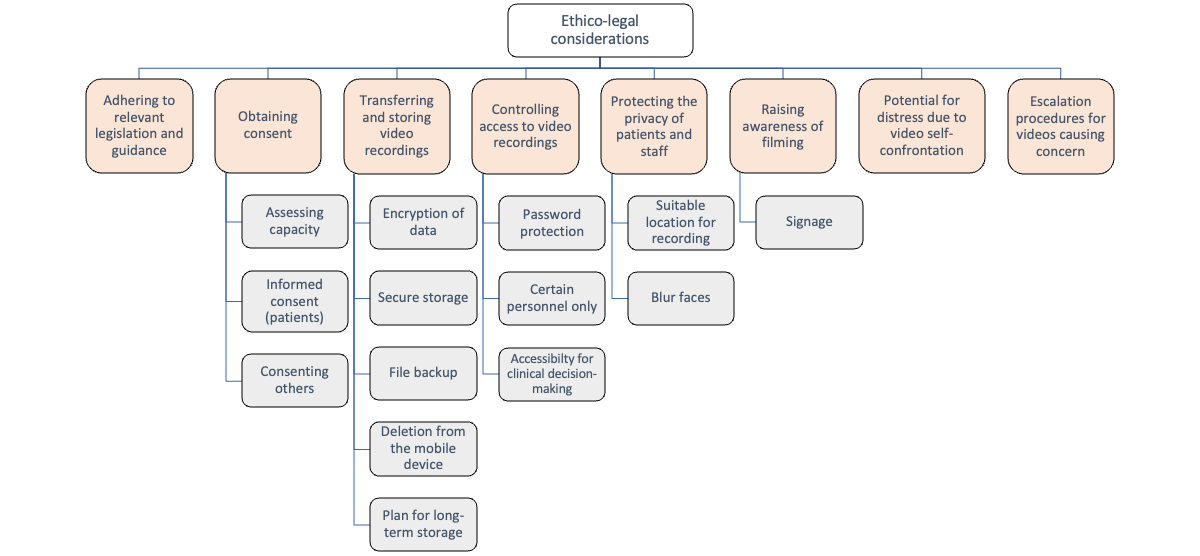
Ethico-legal considerations.

Ethico-legal considerations relating to video recording of patients for direct care purposes were described in all but 3 included studies; these considerations were broadly categorized into 8 themes (Figure 3).

Some authors cited adherence to relevant local, regional, or national legislation and guidelines, including local institutional review boards and clinical information governance protocols, state legislation (US studies), national health service policy, and federal law; for example, the Health Insurance Portability and Accountability Act (1996; US National Standard for Protecting Patient Health Information) [32,38,39,42,45,47]. Key priorities were obtaining informed consent, storage of video recordings, and data protection. Patients consented as principal subjects of the recordings; however, the importance of consenting others who may appear in the video was also raised [48]:

Other people should be kept out of the video as much as possible... but this is probably not possible, so my partner needs to consent as well.

Patient 7

Arrangements for secure transmission and storage of, and access to, video recordings were described, including the use of encryption, secure servers and local network storage devices, password protection and limiting access to certain personnel, file backup, and long-term storage [30,32,34,39,41-43,45,47,51]. Functionality enabling automatic or remote deletion of patient videos from portable recording devices (smartphones and electronic tablets) has also been reported [30,34,45,47]. Although secure storage was considered vital for patient confidentiality, easy access to patients’ video recordings was perceived as equally important for clinical decision-making [54]:

To compare a patient’s examination over time, one ideally would like to have the ability to access a video file quickly and easily in clinic. The desire for accessibility, however, must be balanced by the need for patient privacy and confidentiality.

Results extract

Measures undertaken to protect the privacy of patients and health or care staff included careful consideration given to the video recording location—for example, avoiding placing cameras in bathrooms [42], capturing recordings in a private room [38], and selecting a location where other people are unlikely to unintentionally video record [48]. Further suggestions included the possibility for patients or staff members to stop video recording at any time and use functionality to blur faces [32]. In 2 studies [32,42], the authors described signage (posters or stickers) to raise awareness that video recording was taking place to support care. Escalation procedures for staff members witnessing events causing concern in video recordings were also reported [32]. The authors highlighted the potential for patients to be distressed by seeing themselves on video and highlighted the need to mitigate this possibility when video recordings are viewed by patients as part of their treatment [32,36,38].

#### Guidance of Professional Bodies in the United Kingdom

Several professional bodies in the United Kingdom have provided guidance on or relating to video recording patients for direct care purposes based on national legislation, such as the Data Protection Act 2018 [21], UK General Data Protection Regulation [23], and the Caldicott principles [55] (Textbox 2). Table 4 summarizes the key recommendations of professional bodies, which address many of the ethico-legal considerations raised in the included studies and are consistent with their recommendations, including obtaining informed consent, privacy and confidentiality, data storage, and data protection. Additional recommendations relate to the disclosure of recordings made as part of a patient’s care for secondary use such as research or education and guidance on what to do if a patient wishes to record a clinical consultation.

**Principal legislation and guidance in the United Kingdom.**

**UK General Data Protection Regulation (GDPR) [17]**

- The principal legislation governing how records, information, and personal data are managed, including how personal information may be processed. It is based on seven key principles: (1) lawfulness, fairness, and transparency; (2) purpose limitation; (3) data minimization; (4) accuracy; (5) storage limitation; (6) integrity and confidentiality (security); and (7) accountability.

**Data Protection Act 2018 [15]**

- Sits alongside and supplements the UK GDPR—for example, by providing exemptions.
- Sets out the framework for how personal data must be collected, handled, and stored to protect people’s fundamental right to privacy.
- Supports organizations with their lawful processing of personal data.

**The Caldicott principles [56]**

- Eight principles that all health and social care staff members are expected to adhere to.
  1. Justify the purposes for using confidential information.
  2. Use confidential information only when it is necessary.
  3. Use the minimum necessary confidential information.
  4. Access to confidential information should be on a strict need-to-know basis.
  5. Everyone with access to confidential information should be aware of their responsibilities.
  6. Comply with the law.
  7. The duty to share information for individual care is as important as the duty to protect patient confidentiality.
  8. Inform patients and service users about how their confidential information is used.

**Table 4:** Key recommendations of professional and regulatory bodies.

Professional body	Title of the document and aspects covered	Key recommendations
GMC^a^ (2013) [57]	*Making and using visual and audio recordings of patients^b^* Transparency Consent Confidentiality Data storage and data protection Disclosing recordings	Provide *information* about the *purpose* of the recording Make recordings only with *consent* or other *valid authority* *No unduepressure* to give consent Where practicable, *stop* the recording if the patient asks you to, or if it is having an *adverse effect* on the consultation or treatment Do not make, or participate in making, recordings *against a patient’s wishes*, or where a recording may cause the *patient harm* Do not disclose or use recordings for purposes outside the scope of the original consent without obtaining further consent (except in specific circumstances set out in the GMC Guidance). *Anonymize* or code recordings before using or disclosing them for a secondary purpose, if this is practicable and will serve the purpose *Disclose* or use recordings from which patients may be *identifiable* only with *consent* or other valid authority for doing so *Secure* storage of recordings Follow the *law* and *local guidance* and procedures that apply where you work.
Care Quality Commission (2022) [58]	*Photography and making and using visual recordings of patients* Confidentiality Consent Data storage and data protection	Adhere to recommendations set out in *GMC guidance (2013)* Comply with the *Data Protection Act 2018* Only make recordings for a specific purpose Keep recordings *secure* Ensure recordings are *relevant* and *up to date* Only hold as much information as you need, and *only for as long as you need it*. *Allow the subject of the recording to see it on request* *Store* recordings in an *institutional repository or secure server* (never store recordings on a PC, laptop, USB, or mobile device) Ensure recordings can be traced back to consent Store recordings in the original format, where possible. Ensure files are *backed up regularly* *Avoid* sharing recordings through *social media sites*
National Health Service X (2021) [59]	*Records management code of practice* Clinical relevance Data retention Data storage Transparency	Organizations should decide whether it is *clinically appropriate* to use recordings for direct care. Robust policies should be available. Recordings should be retained in accordance with the retention schedule set out in *Records Management Code of Practice*. Ensure the recording is available throughout the retention period (eg, if a system is becoming obsolete, migrate recording to a newer platform) If stored with a product provider, the organization (as data controller) should provide clear instructions on storage and retention (eg, retain for 8 y from consultation with the patient or service user, then destroy). Explain exactly *how* the recording is to be used, *why*, and *what will happen* with the recording after the interaction.
Royal College of Nursing (2022) [60]	*Patients or family members audio recording or filming in clinical settings*	If a patient or family member is audio recording or filming, then the reasons for this should be discussed. Unless there is good reason for doing so (eg, the patient is unable to recall oral advice or there is a problem with interpreting written material), this action should be stopped.
Royal College of Occupational Therapists (2017) [55]	*Keeping records* Consent Transparency Data storage and data protection Patients making recordings	Obtain *informed consent*—check local policy to see if written consent is required. Store recordings on a secure central system. Do not store recordings on any *portable devices*. If using portable devices, ensure that recordings do not automatically upload to social media or backup sites. *Transfer* of recordings to other clinical staff must be done *securely*. If a friend or family member is taking a recording of a patient, then the reasons for this should be discussed. If there is a *safeguarding concern* or the *confidentiality* of the information is in question, you should stop the interaction and *seek advice* from your employer, or legal advice.
British Medical Association (2021) [61]	*Patients recording consultations*	Allowing a patient to record a consultation to improve recall or understanding of information could be considered as a reasonable adjustment requirement under equality legislation. Explore why the patient wants to make the recording. If you are still unhappy with the encounter being recorded, sensitively explain your reasons to the patient.
British Medical Association (2021) [62]	*Confidentiality toolkit* Disclosing recordings	Recordings made as part of a patient’s care are part of the medical record and are subject to the usual duty of *confidentiality*. These recordings can be shared for the direct care of a patient under *implied consent*. Recordings should be treated in the same way as the rest of the medical record in terms of disclosures for secondary uses (eg, research and education or training); *explicit consent for disclosure will usually be required*.

^a^GMC: General Medical Council.

^b^Title of document is italicized.

## Discussion

### Principal Findings

Video recording patients for direct care purposes appears to be acceptable, and this is evident in the recruitment and retention rates in studies evaluating a video recording intervention and the predominantly positive views and experiences of patients and clinical staff members. Concerns about privacy and physical appearance were voiced by some patients, whereas clinical staff members were concerned about device security, technical issues, and operational and environmental considerations associated with implementing video recording in a clinical setting. Video recordings were perceived to be valuable in supporting patient assessment, care, or treatment; in promoting patient engagement; and in enhancing communication and recall of information. The results of observational studies suggested that video-based patient records are effective in supporting direct care delivery; however, RCTs demonstrated that the video recording intervention was not superior to the controls. Of note, methodological and reporting quality was generally poor considering the studies collectively and worse in observational studies compared with RCTs. This review summarizes and discusses relevant professional guidance and national legislation for UK practice, including recommendations for obtaining consent, protecting privacy and confidentiality, and securing data transmission and storage.

### Comparison With the Wider Literature

Although our review explored video recording in the context of direct care delivery, 2 previous literature reviews examined the evidence on video recording patients’ research and training purposes. A review of audio-visual recordings during general practitioner consultations for research purposes, published 15 years ago in 2008, found that patients who had not been videoed before were more likely to dislike the idea than patients who had previous experience with video recording during health care interactions [63]. In contrast, our findings are consistent with a later 2016 systematic review on the acceptability and design of video-based research, which reported that most patients and clinical staff regard video recording as acceptable and worthwhile for research and training [1]. It follows that the proliferation of smartphones over the last decade, their video recording functionality, and widespread adoption of videoconferencing during the COVID-19 pandemic have led to video recording becoming more acceptable in clinical practice for patients and clinical staff members [11].

Our review found that the factors influencing acceptability among clinical staff were technical issues and mobile device security. Similarly, previous research has reported issues with the installation or usability of video recording equipment, images not being transmitted to patient records, and mobile devices being stolen [12,64,65]. The inherent lack of anonymity in video-based records was reflected in patients’ concerns about their physical appearance or privacy; however, these concerns were voiced by only a small number of patients [31,34,42,48]. However, it is widely recognized that patients are apprehensive about how their personal data are handled and shared in the health care context [66,67]. A lack of transparency in how personal data are used undermines patients’ ability to trust that measures are in place to protect their privacy and thus reduces their willingness to engage with health information technologies [68]. As such, adherence to relevant legislation and professional guidance is not only essential from a medico-legal perspective, but it will also be important for the successful implementation of video-based records within health care settings.

Although video-based records were perceived to enhance several aspects of direct care delivery, the methodological and reporting quality of the body of evidence was generally poor, which limits the conclusions that can be drawn regarding effectiveness. Among the 5 RCTs, video recording interventions were not associated with improvements in clinically relevant outcome measures, although most trials were underpowered. Of note, our study was concerned with direct care delivery, and the applicability of RCTs to real-world clinical practice has been debated [22]. Although the research settings and target populations of the included RCTs were generally well described (Tables S1 and S3 in Multimedia Appendix 4), small sample sizes and lack of discussion around contextual factors that may have influenced the results make it difficult to judge how applicable these trials are to other settings. The results of the included observational studies (arguably more applicable to real-world settings if well designed) favored video recording, but the study designs lacked adjustment for confounders and were not suitable for estimating causal effects. A recent systematic review evaluating home videos for seizures demonstrated consistent improvements in diagnostic accuracy and clinical decision-making [69]. However, some studies in that review did not evaluate video recordings in a real-world direct care delivery context, and similar to our review, the findings were based on studies with diverse patient populations, interventions, and outcome measures that were not designed to test effectiveness, thus limiting internal and external validity.

Pertinent to video recording patients in health care, data protection legislation sets out how personal data must be collected, handled, and stored to protect people’s fundamental right to privacy. Although some countries, such as Canada, have complex data protection legislation at the federal and provincial levels, there is consistency in legislation across the globe pertaining to key issues, such as consent and data security, as laid out in the Personal Information Protection and Electronic Documents Act (2000; Canada) [70], the Data Protection Act (2018; the United Kingdom) [15], General Data Protection Regulations (the United Kingdom and European countries) [16,17], the Health Insurance Portability and Accountability Act (1996; the United States) [18], and the Privacy Act (1988; Australian Privacy Principles) [71]. As professional and regulatory guidelines are underpinned by data protection legislation, it follows that there is consistency in the recommendations for audio-visual recording patients. The professional and regulatory guidance collated for UK practice as part of this review lacks detailed information about how to operationalize the recommendations in clinical practice. Furthermore, there is tension between ensuring patient confidentiality and the accessibility of video recordings to inform clinical decision-making [51]. The Caldicott principles (Textbox 2) underline the need to strike a balance between protecting patient privacy and sharing information responsibly for individual care [56]. Technological advances permitting storage of electronic health records on secure cloud servers, integration of mobile device apps with electronic health records, and secure information exchange between providers will likely support health care organizations in achieving this balance. Although current professional and regulatory guidelines in the United Kingdom make recommendations for securely storing video recordings, they do not consider the importance of recordings being easily accessible to clinicians for direct care purposes.

### Practice Recommendations and Future Research

Ensuring public trust and confidence in how personal data are used by the health service is a central concern for health care organizations and policy makers [72-74]. High levels of patient trust are paramount for the implementation of video-based patient records, particularly given the inherent lack of anonymity in video recordings. Clinical staff involved in delivering video recording interventions must be equipped to provide patients with information on how privacy and confidentiality will be protected [75]. As data controllers, health care organizations should have robust policies and procedures for operationalizing data protection legislation when implementing video-based patient records. This will include arrangements for secure storage and transmission of video recordings and facilities for remote content deletion where portable devices are used.

The methodological and reporting quality of the studies included in this review was generally poor, and further research is needed, particularly for the effectiveness of video-based patient records in direct care delivery. Although RCTs are considered the “gold standard” of causal impact evaluation, they should adopt a pragmatic attitude to evaluating video-based records for direct care purposes and be reported according in line with the CONSORT (Consolidated Standards of Reporting Trials) statement extension for pragmatic trials [76]. Theory-based mixed methods approaches are recommended to identify and explore the active ingredients of video recording practices, including the contextual and behavioral factors driving possible effects [77].

### Strengths and Limitations

A strength of this review lies in the comprehensive synthesis of qualitative and quantitative data and professional guidance to address a range of important considerations for the implementation of video-based patient records. A robust systematic review process was followed, with 2 reviewers independently undertaking screening, data extraction, quality appraisal, and coding of qualitative findings. We ensured rigor and transparency in the qualitative synthesis by applying the framework method to derive insights from the included studies [20]. However, the number of studies on video recording for direct care purposes is small and scattered across traditional disease boundaries. Therefore, despite our efforts to carefully perform the search and selection processes, we may have missed some relevant studies. As it was anticipated that the body of literature would be small, we included studies with different designs and did not exclude low-quality studies. Heterogeneity relating to the study populations, video recording interventions, and outcome measures as well as the collectively poor methodological and reporting quality of the studies mean that firm conclusions cannot be drawn, particularly in relation to whether video-based patient records are effective in supporting direct care delivery. This review sought to collate professional and regulatory guidance for video recording patients in the British National Health Service. Although these recommendations may not directly translate to health care settings outside the United Kingdom, there is broad consistency in data protection legislation around the globe regarding several key issues, such as seeking consent and implementing data security measures.

### Conclusions

Video technologies have been piloted in various health and care contexts to support diagnosis, care, and treatment. Video recording practices appear to be acceptable to patients and clinical staff despite concerns about privacy, technical considerations, and integration into clinical workflows. The methodological and reporting limitations of the studies included in this review prevented firm conclusions from being drawn. Pragmatic, mixed methods trials, particularly in the field of movement disorders, older adult care, and in patients’ homes, should evaluate how video recording impacts diagnosis and treatment monitoring, patient and care provider communication, and patient safety. Professional and regulatory documents should provide practical guidance for the secure, ethical implementation of video recording in routine practice.

## Appendix

Multimedia Appendix 1 Detailed search strategy.

Multimedia Appendix 2 PRISMA (Preferred Reporting Items for Systematic Reviews and Meta-Analyses) checklist.

Multimedia Appendix 3 Key characteristics of included studies.

Multimedia Appendix 4 Quality assessment of included studies.

Multimedia Appendix 5 Recruitment and retention rates as measures of video recording intervention acceptability.

Multimedia Appendix 6 Acceptability and perceived effectiveness of video recording patients for direct care purposes: subthemes and additional verbatim quotes.

## Abbreviations

CONSORT: Consolidated Standards of Reporting Trials
PRISMA: Preferred Reporting Items for Systematic Reviews and Meta-Analyses
QuADS: Quality Assessment with Diverse Studies
RCT: randomized controlled trial
STROBE: Strengthening the Reporting of Observational Studies in Epidemiology
TFA: Theoretical Framework of Acceptability

## References

[1] Parry R, Pino M, Faull C, Feathers L (2016). Acceptability and design of video-based research on healthcare communication: evidence and recommendations. Patient Educ Couns.

[2] Mazer L, Varban O, Montgomery JR, Awad MM, Schulman A (2022). Video is better: why aren't we using it? A mixed-methods study of the barriers to routine procedural video recording and case review. Surg Endosc.

[3] Baumann SE, Merante M, Folb BL, Burke JG (2020). Is film as a research tool the future of public health? A review of study designs, opportunities, and challenges. Qual Health Res.

[4] Latvala E, Vuokila-Oikkonen P, Janhonen S (2000). Videotaped recording as a method of participant observation in psychiatric nursing research. J Adv Nurs.

[5] van de Graaf FW, Lange MM, Spakman JI, van Grevenstein WM, Lips D, de Graaf EJ, Menon AG, Lange JF (2019). Comparison of systematic video documentation with narrative operative report in colorectal cancer surgery. JAMA Surg.

[6] Dimick JB, Scott JW (2019). A video is worth a thousand operative notes. JAMA Surg.

[7] Pedersen CG, Høstrup LH, Gadager BB, Nielsen CV, Maribo T, Madsen LS (2022). Understanding healthcare providers' experiences with video recording of patient consultations. Prim Health Care Res Dev.

[8] Kim DE, Sagong H, Kim E, Jang AR, Yoon JY (2019). A systematic review of studies using video-recording to capture interactions between staff and persons with dementia in long-term care facilities. J Korean Acad Community Health Nurs.

[9] Kripalani S, LeFevre F, Phillips CO, Williams MV, Basaviah P, Baker DW (2007). Deficits in communication and information transfer between hospital-based and primary care physicians: implications for patient safety and continuity of care. JAMA.

[10] Warren LR, Clarke J, Arora S, Darzi A (2019). Improving data sharing between acute hospitals in England: an overview of health record system distribution and retrospective observational analysis of inter-hospital transitions of care. BMJ Open.

[11] Manojlovich M, Frankel RM, Harrod M, Heshmati A, Hofer T, Umberfield E, Krein S (2019). Formative evaluation of the video reflexive ethnography method, as applied to the physician-nurse dyad. BMJ Qual Saf.

[12] Taylor-Gjevre R, Nair B, Bath B, Okpalauwaekwe U, Sharma M, Penz E, Trask C, Stewart SA (2018). Addressing rural and remote access disparities for patients with inflammatory arthritis through video-conferencing and innovative inter-professional care models. Musculoskeletal Care.

[13] Funkenstein AB, Kessler KA, Schen CR (2014). Learning through the lens: ethical considerations in videotaping psychotherapy. Harv Rev Psychiatry.

[14] Alpert MC (1996). Videotaping psychotherapy. J Psychother Pract Res.

[15] Data Protection Act 2018. Legislation.gov.uk.

[16] (2018). Reform of EU data protection rules. European Commission.

[17] UK GDPR guidance and resources. Information Commissioner's Office.

[18] (1996). Health information privacy. U.S. Department of Health and Human Services.

[19] Using and disclosing patient information for direct care. General Medical Council.

[20] Dixon-Woods M (2011). Using framework-based synthesis for conducting reviews of qualitative studies. BMC Med.

[21] Sekhon M, Cartwright M, Francis JJ (2017). Acceptability of healthcare interventions: an overview of reviews and development of a theoretical framework. BMC Health Serv Res.

[22] Treweek S, Zwarenstein M (2009). Making trials matter: pragmatic and explanatory trials and the problem of applicability. Trials.

[23] Harrison R, Jones B, Gardner P, Lawton R (2021). Quality assessment with diverse studies (QuADS): an appraisal tool for methodological and reporting quality in systematic reviews of mixed- or multi-method studies. BMC Health Serv Res.

[24] Joanna Briggs Institute homepage. Joanna Briggs Institute.

[25] Vandenbroucke JP, von Elm E, Altman DG, Gøtzsche PC, Mulrow CD, Pocock SJ, Poole C, Schlesselman JJ, Egger M, STROBE Initiative (2007). Strengthening the Reporting of Observational Studies in Epidemiology (STROBE): explanation and elaboration. PLoS Med.

[26] Liberati A, Altman DG, Tetzlaff J, Mulrow C, Gøtzsche PC, Ioannidis JP, Clarke M, Devereaux PJ, Kleijnen J, Moher D (2009). The PRISMA statement for reporting systematic reviews and meta-analyses of studies that evaluate healthcare interventions: explanation and elaboration. BMJ.

[27] David AS, Chis Ster I, Zavarei H (2012). Effect of video self-observations vs. observations of others on insight in psychotic disorders. J Nerv Ment Dis.

[28] Kenny M, Gilmartin J, Thompson C (2022). Video-guided exercise after stroke: a feasibility randomised controlled trial. Physiother Theory Pract.

[29] Jayabalan P, Kaplan R, Breisinger T, Greene E, Vitti K, Lanphere J, Shen J (2014). Poster 311 video recording the gait of stroke patients during inpatient rehabilitation to improve motivation, satisfaction and outcome. PM&R.

[30] Sharma S, McCrary H, Romero E, Kim A, Chang E, Le CH (2018). A prospective, randomized, single-blinded trial for improving health outcomes in rhinology by the use of personalized video recordings. Int Forum Allergy Rhinol.

[31] Schandrin A, Picot M-C, Marin G, André M, Gardes J, Léger A, O'Donoghue B, Raffard S, Abbar M, Capdevielle D (2022). Video self-confrontation as a therapeutic tool in schizophrenia: a randomized parallel-arm single-blind trial. Schizophr Res.

[32] Bayen E, Jacquemot J, Netscher G, Agrawal P, Tabb Noyce L, Bayen A (2017). Reduction in fall rate in dementia managed care through video incident review: pilot study. J Med Internet Res.

[33] du Mortier JA, Visser HA, van Geijtenbeek-de Vos van Steenwijk MF, van Megen HJ, van Balkom AJ (2019). Use of videotaped personal compulsions to enhance motivation in obsessive-compulsive disorder. BJPsych Open.

[34] Garfein RS, Collins K, Muñoz F, Moser K, Cerecer-Callu P, Raab F, Rios P, Flick A, Zúñiga ML, Cuevas-Mota J, Liang K, Rangel G, Burgos JL, Rodwell TC, Patrick K (2015). Feasibility of tuberculosis treatment monitoring by video directly observed therapy: a binational pilot study. Int J Tuberc Lung Dis.

[35] Okuyama S, Fischer S, Bekelman D (2014). Implementing and evaluating video dignity therapy in an underserved and ethnically diverse advanced cancer patient population. Support Care Cancer.

[36] Quintiliani LM, Waite K, Volandes AE, Paasche-Orlow MK (2020). Acceptability of video declarations of advance care planning preferences by cancer patients. J Gen Intern Med.

[37] Quintiliani LM, Murphy JE, Buitron de la Vega P, Waite KR, Armstrong SE, Henault L, Volandes AE, Paasche-Orlow MK (2018). Feasibility and patient perceptions of video declarations regarding end-of-life decisions by hospitalized patients. J Palliat Med.

[38] Towsley GL, Wong B, Mokhtari T, Hull W, Miller SC (2020). Piloting me and my wishes-videos of nursing home residents' preferences. J Pain Symptom Manage.

[39] Williams K, Arthur A, Niedens M, Moushey L, Hutfles L (2013). In-home monitoring support for dementia caregivers: a feasibility study. Clin Nurs Res.

[40] Dash D, Singh R, Vibha D, Tripathi M (2020). Home videos made on smart phone supplements paper based diary for correct identification of motor states in Parkinson’s disease. Mov Disord.

[41] Amin U, Primiani CT, MacIver S, Rivera-Cruz A, Frontera AT, Benbadis SR (2021). Value of smartphone videos for diagnosis of seizures: everyone owns half an epilepsy monitoring unit. Epilepsia.

[42] Bayen E, Nickels S, Xiong G, Jacquemot J, Subramaniam R, Agrawal P, Hemraj R, Bayen A, Miller BL, Netscher G (2021). Reduction of time on the ground related to real-time video detection of falls in memory care facilities: observational study. J Med Internet Res.

[43] Ojeda J, Gutierrez G, Villegas RD, Ivañez V, López Gallardo S (2016). Utility of home-made videos in an adult epilepsy clinic. J Neurol Disord.

[44] Dash D, Sharma A, Yuvraj K, Renjith A, Mehta S, Vasantha PM, Arora A, Tripathi M (2016). Can home video facilitate diagnosis of epilepsy type in a developing country?. Epilepsy Res.

[45] Guthrie S, Stansfield J (2020). Dysphagia assessment and intervention: evaluating inclusive approaches using video. Adv Ment Health Intellect Disabil.

[46] Naeem K, Bhargava M, Porter RW (2022). Trends of in-patient use of patient-provider video recording during COVID19 era and its effects on HCAHPS scores. Neurosurgery.

[47] Meeusen AJ, Porter R (2015). Patient-reported use of personalized video recordings to improve neurosurgical patient-provider communication. Cureus.

[48] de Vries NM, Smilowska K, Hummelink J, Abramiuc B, van Gilst MM, Bloem BR, de With PH, Overeem S (2019). Exploring the Parkinson patients' perspective on home-based video recording for movement analysis: a qualitative study. BMC Neurol.

[49] Davin P (2015). The use of a cost-effective high speed video camera to assess abnormal gait mechanics in an avid runner with lumbosacral dysfunction. ProQuest.

[50] Freund B, Tatum WO (2021). Pitfalls using smartphones videos in diagnosing functional seizures. Epilepsy Behav Rep.

[51] Duker AP (2013). Video recording in movement disorders: practical issues. Continuum (Minneap Minn).

[52] Rocha J, Pereira J (2013). Importance of video technologies in seizure identification – evidence through a case report. Int J Case Rep Med.

[53] Singh M, Benjamin M-J, Turenne M, Lang G, Li H, Bueno E, Carty MJ, Pribaz JJ, Pomahac B, Talbot SG (2015). Use of video clips to assess the outcomes of bilateral hand transplantation. Plast Reconstr Surg Glob Open.

[54] Duker AP (2013). Video recording in movement disorders: practical issues. Continuum (Minneap Minn).

[55] Royal College of Occupational Therapists (2017). Keeping Records Guidance for occupational therapists.

[56] (2020). The Caldicott principles. National Data Guardian United Kingdom Government.

[57] General Medical Council (2002). Making and using visual and audio recordings of patients (May 2002). J Audiov Media Med.

[58] (2022). GP mythbuster 62: photography and making and using visual recordings of patients. Care Quality Commission.

[59] (2021). Records management code of practice 2021: a guide to the management of health and care records. NHSx.

[60] (2022). Audio recording or filming in clinical settings by patient or third party. Royal College of Nursing.

[61] (2021). Patients recording consultations. British Medical Association.

[62] (2021). Confidentiality toolkit. British Medical Association.

[63] Themessl-Huber M, Humphris G, Dowell J, Macgillivray S, Rushmer R, Williams B (2008). Audio-visual recording of patient-GP consultations for research purposes: a literature review on recruiting rates and strategies. Patient Educ Couns.

[64] Gonçalves-Bradley DC, J Maria AR, Ricci-Cabello I, Villanueva G, Fønhus MS, Glenton C, Lewin S, Henschke N, Buckley BS, Mehl GL, Tamrat T, Shepperd S (2020). Mobile technologies to support healthcare provider to healthcare provider communication and management of care. Cochrane Database Syst Rev.

[65] Chang LW, Kagaayi J, Arem H, Nakigozi G, Ssempijja V, Serwadda D, Quinn TC, Gray RH, Bollinger RC, Reynolds SJ (2011). Impact of a mHealth intervention for peer health workers on AIDS care in rural Uganda: a mixed methods evaluation of a cluster-randomized trial. AIDS Behav.

[66] Entzeridou E, Markopoulou E, Mollaki V (2018). Public and physician's expectations and ethical concerns about electronic health record: benefits outweigh risks except for information security. Int J Med Inform.

[67] Small SS, Hohl CM, Balka E (2021). Patient perspectives on health data privacy and implications for adverse drug event documentation and communication: qualitative study. J Med Internet Res.

[68] LaMonica HM, English A, Hickie IB, Ip J, Ireland C, West S, Shaw T, Mowszowski L, Glozier N, Duffy S, Gibson AA, Naismith SL (2017). Examining internet and ehealth practices and preferences: survey study of Australian older adults with subjective memory complaints, mild cognitive impairment, or dementia. J Med Internet Res.

[69] Ricci L, Boscarino M, Assenza G, Tombini M, Lanzone J, Di Lazzaro V, Casciato S, D'Aniello A, Morano A, Di Gennaro G, Epilepsy Study Group of the Italian Neurological Society (2021). Clinical utility of home videos for diagnosing epileptic seizures: a systematic review and practical recommendations for optimal and safe recording. Neurol Sci.

[70] (2000). Personal information protection and electronic documents act (S.C. 2000, c. 5). Government of Canada.

[71] (1988). Privacy act 1988. Australian Government.

[72] Gille F, Smith S, Mays N (2022). Evidence-based guiding principles to build public trust in personal data use in health systems. Digit Health.

[73] (2021). Data saves lives: reshaping health and social care with data (draft). Department of Health and Social Care United Kingdom Government.

[74] Goldacre B, Morley J (2022). Better, broader, safer: using health data for research and analysis. Department of Health and Social Care.

[75] Gille F, Brall C (2021). Limits of data anonymity: lack of public awareness risks trust in health system activities. Life Sci Soc Policy.

[76] Zwarenstein M, Treweek S, Gagnier JJ, Altman DG, Tunis S, Haynes B, Oxman AD, Moher D, CONSORT group, Pragmatic Trials in Healthcare (Practihc) group (2008). Improving the reporting of pragmatic trials: an extension of the CONSORT statement. BMJ.

[77] Skivington K, Matthews L, Simpson SA, Craig P, Baird J, Blazeby JM, Boyd KA, Craig N, French DP, McIntosh E, Petticrew M, Rycroft-Malone J, White M, Moore L (2021). A new framework for developing and evaluating complex interventions: update of Medical Research Council guidance. BMJ.

