# Supplementary material for: Video Recording Patients for Direct Care Purposes: Systematic Review and Narrative Synthesis of International Empirical Studies and UK Professional Guidance
Source: J Med Internet Res. 2023 Aug 16;25:e46478. doi: 10.2196/46478 (PMC10468707; doi:10.2196/46478)
Supplement: Multimedia Appendix 1 [file jmir_v25i1e46478_app1.docx]

**Multimedia Appendix 1**. Detailed search strategy.

| **Set** | **Search Statement** |
| --- | --- |
| 1. | filmed.ab,ti. |
| 2. | filming.ab,ti. |
| 3. | video record*.ab,ti. |
| 4. | video-record*.ab,ti. |
| 5. | video clip*.ab,ti. |
| 6. | video footage.ab,ti. |
| 7. | videotap*.ab,ti. |
| 8. | visual record*.ab,ti. |
| 9. | patient*.ab,ti. |
| 10. | service user*.ab,ti. |
| 11. | service-user.ab,ti. |
| 12. | inpatient*.ab,ti. |
| 13. | outpatient*.ab,ti. |
| 14. | resident*.ab,ti. |
| 15. | 1 or 2 or 3 or 4 or 5 or 6 or 7 or 8 |
| 16. | 9 or 10 or 11 or 12 or 13 or 14 |
| 17. | 15 and 16 |
| 18. | limit 17 to (english language and yr="2012 -Current") |

Last search performed on 13^th^ December 2022.
