# Supplementary material for: Video Recording Patients for Direct Care Purposes: Systematic Review and Narrative Synthesis of International Empirical Studies and UK Professional Guidance
Source: J Med Internet Res. 2023 Aug 16;25:e46478. doi: 10.2196/46478 (PMC10468707; doi:10.2196/46478)
Supplement: Multimedia Appendix 3 [file jmir_v25i1e46478_app3.docx]

**Multimedia Appendix 3.** Key characteristics of included studies

| Author (Year)  Country | Study aim(s)  Study Design | Target Population  Sample size | Intervention  (Comparator) | Outcomes |
| --- | --- | --- | --- | --- |
| Video recording to support diagnosis(a) | | | | |
| Guthrie  (2020)  UK | To explore and evaluate the use of video clips in dysphagia assessment and intervention from the perspective of service users, ward staff and Speech & Language Therapists (SLT)  Prospective observational | Inpatients with intellectual disabilities and mental health conditions with suspected dysphagia  N=44 | Materials & procedures:  iPad; video clips of mealtimes were recorded jointly by the SLTs and service-users to assess service-user presentation when drinking and eating. Videoclips were also taken to capture facial tone and movements. Service-users, ward staff and SLTs reviewed the video clips and provided comments/feedback.  Who viewed? Service users, ward staff, SLTs  Setting: Long-term residential (inpatient) accommodation, including medium and low secure forensic wards.  (No comparator) | - Practical issues for using iPad videos  - Potential for direct use in dysphagia assessment and interventions |
| Dash  (2020)(b)  India | To assess feasibility of home videos made on smart phone as a supplemental tool to paper-based diary in registering motor states.  Feasibility RCT | Patients with Parkinson’s disease  N=128 | Materials & procedures:  Smartphones; patients & caregivers provided with explanations about the various motor states (OFF, ON, dyskinesia). Home videos of various states taken, timing recorded in corresponding diary entry. Diary entries & videos checked to determine motor states.  Who viewed? Clinicians  Setting: Patients’ home.  (Paper-based diary completed by patient/caregiver) | - Quality of home videos  - Accuracy in correctly identifying motor states |
| De Vries  (2019)  The Netherlands | To explore the patient’s opinion on home-based video recording using vision-based movement analysis  Qualitative Grounded Theory | Patients with Parkinson’s disease  N=16 | Materials & procedures:  Kinect camera - 3D depth camera; continuous video recording and measurement of motor function.  Who viewed? Not reported  Setting: Patient’s home  (No comparator) | - Acceptability of home video recording |
| Duker  (2013)  US | To describe issues surrounding the practical application of video recording in the clinical setting  Case Report | Patient with Parkinson’s disease  N=1 | Materials & procedures:  Not reported; video recordings taken during initial assessment and in follow up to enable evaluation of the patient’s movements.  Who viewed? Clinicians  Setting: Movement disorder center  (No comparator) | - Utility of video in assessment of movement disorder  - Practical issues associated with video recordings |
| Amin  (2021)  US | To evaluate the value of smartphone videos for the diagnosis of seizures.  Prospective observational | Patients presenting with seizures  N=54 | Materials & procedures:  Smartphones; seizure video recorded by caregiver.  Who viewed? Board-certified epileptologists  Setting: Not reported.  (Usual care: video EEG) | - Accuracy of smartphone videos in distinguishing between epileptic & non-epileptic events |
| Dash  (2016)  India | To evaluate the feasibility and yield of semiological features from home videos and compare them to those inferred from history provided by the caregiver of a person with epilepsy.  Prospective observational | Patients presenting with seizures  N=340 | Materials & procedures:  Smartphones; caregiver makes home videos of patients’ seizures. Video quality assessed. Diagnosis made based on home video.  Who viewed? Epileptologists  Setting: Patients’ home.  (Usual care: video EEG & caregiver history) | - Quality of home videos  - Accuracy of semiological signs inferred from videos compared to i) caregiver history ii) video EEG |
| Freund  (2021)  US | To illustrate the pitfalls in using video recordings obtained by patients and family members when used as an adjunct to evaluate people with seizures.  Case Report | Patient presenting with seizures  N=1 | Materials & procedures:  Smartphone; wife had recorded a video of patient's habitual seizures  Who viewed?  Epileptologist  Setting:  Patient’s home  (Usual care: video EEG) | - Pitfalls of using patient-recorded smartphone videos alone as a diagnostic tool |
| Ojeda  (2012)  Spain | To assess the feasibility of asking patients with epilepsy and/or caregivers to record home videos during seizures and assess the utility of these recordings in epilepsy diagnosis confirmation  Prospective observational | Patients with drug-resistant epilepsy  N=132 | Materials & procedures:  Any available devices: phone, webcam, video camera; patients encouraged to make home video recordings of their seizures. Videos reviewed during clinical sessions and diagnosis was made.  Who viewed? Neurologists & epileptologists  Setting: Adult Epilepsy Outpatient Clinic  (Previous diagnosis obtained from historical clinical records) | - Quality of video recordings  - Number of confirmed diagnoses & misdiagnoses |
| Rocha  (2013)  Portugal | To alert the medical community to the usefulness of widespread portable video technologies in the diagnosis of seizure disorders.  Case Report | Patient presenting with seizures  N=1 | Materials & procedures:  Smartphone; patient self-recorded a seizure: after experiencing epigastric aura, patient activated the camera recording function and place the smartphone on a desk pointing at himself.  Who viewed? Not reported  Setting: Video taken in patient’s own home; viewed in a hospital neurology department  (CT scan, EEG, video EEG all normal - patient was asymptomatic during routine investigations) | - Usefulness of home video in diagnosing epilepsy |
| Video recording to support care(a) | | | | |
| Bayen  (2017)  US | To analyse how continuous video monitoring and review of falls of individuals with dementia can better support quality of care.  Intervention-only pilot study | Care home residents with dementia  N=38 | Materials & procedures:  Wall-mounted cameras in common areas & in private rooms of consenting residents; continuous video monitoring. Videos of falls events reviewed via a customised mobile application.  Who viewed? Residential home staff  Setting: Residential memory care facility.  (No comparator) | - Falls review utilisation by facility staff  - Rate of falls  - Fall risk patterns |
| Bayen  (2021)  US | To examine the effect of a new technology called SafelyYou Guardian on early post-fall care.  Prospective observational | Care home residents with dementia  N=66 | Materials & procedures:  Wall-mounted cameras in common areas & in private rooms of consenting residents; a fall incident was detected by the AI-enabled camera system. Facility staff received real-time notifications of falls events.  Who viewed? Residential home staff  Setting: Six residential memory care facilities.  (No comparator) | - Length of time until staff assistance arrives  - Length of time on the ground |
| Towsley  (2020)  US | To evaluate the feasibility and acceptability of Me & My Wishes -person-centered videos of residents discussing their preferences for daily and end-of-life (EOL) care.  Intervention-only descriptive pilot study | Long-term nursing home residents  N=20 | Materials & procedures:  Not reported; conversations with patients about their care preferences for daily and end-of-life care were video recorded. Videos were viewed during care conferences.  Who viewed? Patients, their family members, nursing home staff  Setting: Three nursing homes  (No comparator) | - Feasibility  - Acceptability |
| Williams  (2013)  US | To explore feasibility of in-home video monitoring and feedback to help caregivers and reduce caregiving burden.  Feasibility-testing with first caregiver-patient dyad | Patient with Alzheimer’s disease and their caregiver  N=1 caregiver-patient dyad | Materials & procedures:  Laptop computer, high-def webcam. Telehealth application/ Behavior Capture program: a ‘go-back-in time’ digital system, which saves up to 30 min of video recording for review prior to the trigger activation to capture events leading up to a behavior; an expert home and telehealth nurse made home visits to set up the computer and train the caregiver to use the system. The caregiver triggered video recordings when faced with loved one’s challenging behaviours.  Who viewed? Video recordings were automatically uploaded for interdisciplinary team review and feedback  Setting: Patient’s home  (No comparator) | - Acceptability  - Feasibility  - Change in caregiver burden |
| Meeusen  (2015)  US | To describe patient and provider experiences of video recording clinical medical encounters and providing the patient with a copy of the video for informational purposes.  Cross-sectional survey | Patients visiting a neurosurgeon  N=333 | Materials & procedures:  Pre 2014: handheld Sony camera; after 2014: Nexus 7 tablet computers with mobile app; patients were video recorded during their neurosurgical encounter and were provided access to the recording to watch as a way to recall what the doctor said.  Who viewed? Patients  Setting: Neurosurgical clinic  (No comparator) | - Recall, comprehension, stress & anxiety.  - Acceptability  - Technical issues |
| Naeem(b)  (2022)  US | To analyse change in Hospital Consumer Assessment of Healthcare Providers and Systems (HCAHPs) scores associated with patient video recordings during Covid-19 visitation restrictions  Retrospective observational | Hospital inpatients & their families  N not reported | Materials & procedures:  Not reported; video recordings taken of the patients’ and healthcare provider’s interactions and daily progress. Recordings made available to patient and their family members.  Who viewed? Patients & their family members  Setting: Hospital  (No comparator) | - Change in HCAHPs scores  - Experiences of patients and families |
| Okuyama(b) (2014)  US | To assess the feasibility and patient satisfaction of video Dignity Therapy in ethnically diverse advanced cancer patients  Intervention-only pilot study | Patients with stage IV cancer  N=13 | Materials & procedures:  Not reported; Dignity Therapy is a meaningful intervention to address existential suffering for those facing advanced illness. The Dignity Therapy interview with trained personnel is video recorded.  Who viewed? Patients receive a transcript of the interview that is a legacy gift to share with loved ones.  Setting: Safety-net hospital  (No comparator) | - Feasibility  - Satisfaction |
| Quintiliani(b)  (2020)  US | To examine the acceptability of video declarations among patients with advance cancer from the patient's perspective.  Intervention-only pilot study | Patients with advanced cancer  N=29 | Materials & procedures:  iPad; educational video; after viewing a brief educational video describing approaches to end-of-life care, a tablet computer was used to video record patients as they described their wishes for medical care including CPR/intubation.  Who viewed? Patients – to verify that the video accurately represented their wishes  Setting: Outpatient & inpatient settings of a large urban safety-net hospital  (No comparator) | - Acceptability |
| Quintiliani  (2018)  US | To evaluate the content and acceptability of video declarations describing patients’ advance care planning preferences  Intervention-only pilot study | Hospital inpatients (medical conditions not reported)  N=16 | Materials & procedures:  iPad; educational video; patients shown a brief video describing three approaches to end-of-life care. Patients then videotape themselves making a declaration about their wishes.  Who viewed? Patients – to verify that the video accurately represented their wishes.  Setting: Large urban safety-net hospital  (No comparator) | - Acceptability  - Content of video declarations |
| Video recording to support treatment(a) | | | | |
| David  (2012)  UK | To test the hypothesis that schizophrenia patients improve their insight after viewing videos of themselves when unwell more so than after viewing an actor.  RCT | Hospital inpatients with acute psychotic disorders  N=40 | Materials & procedures:  Portable digital video camera; DVD recordings; interview with research psychiatrist video recorded.  Patient shown video recording of ‘self’.  Who viewed? Patients & psychiatrists  Setting: Inpatient psychiatry unit  (Patient shown pre-recorded control interview of an actor playing the part of someone with acute psychosis) | - Change in insight into illness & treatment |
| Schandrin  (2022)  France | To assess the effect of video self-confrontation as a therapeutic tool.  RCT | Inpatients with schizophrenia and acute psychosis  N=60 | Materials & procedures:  Materials not reported. Patients’ acute psychotic episodes were filmed on admission. Patients randomized to the experimental group watched their video footage.  Who viewed? Patients  Setting: Two psychiatric hospitals | - Patient insight into psychotic behaviour  - Psychotic & depressive symptoms  - Medication adherence  - Functioning |
| DuMortier  (2019)  The Netherlands | To describe how patients watching videotaped personal compulsions influences motivation for treatment and whether patients report any adverse events  Intervention-only pilot study | Inpatients with Obsessive Compulsive Disorder  N=26 | Materials & procedures:  Not reported; selected compulsions video recorded. Patients watch videos during regular inpatient treatment. Semi-structured interviews to discover the participants' experiences of watching their videos.  Who viewed? Patients & psychiatrists  Setting: Video recordings taken in patients’ homes; video recordings watched during an inpatient Exposure & Response Prevention treatment programme  (No comparator) | - Patients’ responses to watching home videos of their personal compulsive rituals  - Effect on motivation for treatment  - Adverse events |
| Davin  (2016)(b)  US | To demonstrate the use of a high-speed camera to analyse the gait pattern of a runner presenting with lumbosacral dysfunction.  Case report | Patient with lumbosacral dysfunction  N=1 | Materials & procedures:  High-speed camera; treadmill; patient’s gait video recorded whilst running on a treadmill. Frame-by-frame slow motion analysis. Verbal & visual feedback using the videos to assist the patient in attaining the desired forefoot strike pattern.  Who viewed? Patient & physiotherapist  Setting: Physical therapy clinic  (No comparator) | - Recognition of subtle gait deficits  - Patient’s experience of visual feedback with videos |
| Garfein  (2015)  US; Mexico | To evaluate the feasibility and acceptability of Video Directly Observed Therapy  (V-DOT)  Intervention-only pilot study | Patients receiving treatment for pulmonary tuberculosis (TB)  N=52 | Materials & procedures:  Smartphone application for securely recording, transferring, & storing videos; web-based client management system used by DOT workers to view and document each event; patients record videos of themselves taking each dose of TB medication. Uploaded videos are watched by DOT workers via a secure website to document whether the complete dose was ingested. Daily text message reminders before medication due and after video received.  Who viewed? Health care providers  Setting: Public health departments in high & low resource settings  (No comparator) | - Medication adherence rate  - Patients’ V-DOT experiences |
| Jayabalan(b)  (2014)  US | To improve the motivation, satisfaction, and functional outcome of stroke patients by showing them standardised recordings of their gait and graphical representations of their progress during their rehabilitation stay.  RCT | Inpatients undergoing rehabilitation following acute stroke  N=23 | Materials & procedures:  Not reported; patients’ gait was video recorded performing the Timed Up and Go (TUG) test and the 10-meter walk test. Patients viewed their video recordings taken since admission during weekly counselling sessions. Patients were also shown graphical representations of change in the TUG and 10-meter walk test.  Who viewed? Patients & physicians  Setting: Inpatient stroke rehabilitation unit in a tertiary referral center  (Patients not being video recorded) | - Motivation  - Satisfaction  - Change in TUG |
| Kenny  (2020)  UK | To investigate the feasibility and acceptability of video guided exercise for facilitating upper-limb exercise after stroke.  Feasibility RCT | Inpatients newly diagnosed with stroke  N=14 | Materials & procedures:  Mobile tablets; patients had out-of-therapy-time exercises provided as video on a mobile tablet. Exercises were filmed during the participants' normal therapy sessions. Therapists were filmed performing exercises followed by the patient performing the exercises. Therapists provided verbal prompts to help correct movements. Patients were encouraged to watch exercise videos three times before practising themselves.  Who viewed? Patients  Setting: Two stroke units and a general neuro-rehabilitation ward in a large teaching hospital  (Usual care: verbal or written exercise instructions) | - Acceptability & experience of using tablet-guided exercise therapy  - Change in impairment & disability |
| Sharma  (2018)  US | To evaluate if patient recall, understanding of the disease process and surgery, and patient satisfaction improved with access to the video recordings.  RCT | Patients undergoing functional endoscopic sinus surgery (FESS)  N=101 | Materials & procedures:  The Medical Memory (TMM) devices: Nexus 7 tablet computers loaded with proprietary mobile app; videos were recorded at the end of each preoperative clinical visit and immediate postoperative meeting with the family. Patients then randomized to be given or denied access to the video recording via the cloud server.  Who viewed? Patients & their families  Setting: Tertiary Rhinology Clinic  (Patients without access to video recordings of clinical encounters) | - Accuracy of patient recall of surgical risks  - Patient satisfaction |
| Singh  (2015)  US | To assess the role of video clips in assessing the outcomes of bilateral hand transplantation.  Case Report | Index patient undergoing hand transplantation  N=1 | Materials & procedures:  Not reported; video clips capturing functional progress/outcomes taken at standardised time points in follow up. The patient also sent in multiple home-video clips demonstrating new aspects of function when these improvements occurred.  Who viewed? Patient & clinicians  Setting: Patient’s home & hospital clinic  (No comparator) | - Functional outcomes  - Performance of Activities of Daily Living |

(a)Direct care defined activities that directly contribute to the diagnosis, care, or treatment of an individual [19]. (b)Conference abstract; (c)Doctoral thesis; (all other citations are full journal articles). RCT: Randomised Controlled Trial. N/A: Not Applicable.
