# Supplementary material for: Video Recording Patients for Direct Care Purposes: Systematic Review and Narrative Synthesis of International Empirical Studies and UK Professional Guidance
Source: J Med Internet Res. 2023 Aug 16;25:e46478. doi: 10.2196/46478 (PMC10468707; doi:10.2196/46478)
Supplement: Multimedia Appendix 4 [file jmir_v25i1e46478_app4.docx]

**Multimedia Appendix 4**. Quality assessment of included studies.

Table S1. Quality assessment of included studies: all study designs (excluding case reports)

| **Quality Assessment with Diverse Studies (QuADS) appraisal criteria^(a)^** | Amin (2021) | Bayen  (2017) | Bayen (2021) | Dash (2016) | David (2012) | DeVries  (2019) | Du Mortier  (2019) | Garfein  (2015) | Guthrie (2020) |
| --- | --- | --- | --- | --- | --- | --- | --- | --- | --- |
| 1. Theoretical or conceptual underpinning to the research | 2 | 3 | 3 | 2 | 3 | 3 | 3 | 3 | 3 |
| 2. Statement of research aim/s | 3 | 3 | 3 | 3 | 3 | 2 | 3 | 3 | 3 |
| 3. Clear description of research setting and target population | 2 | 3 | 2 | 2 | 2 | 2 | 3 | 3 | 3 |
| 4. The study design is appropriate to address the stated research aim/s | 3 | 3 | 2 | 3 | 3 | 3 | 3 | 3 | 3 |
| 5. Appropriate sampling to address the research aim/s | 1 | 2 | 1 | 1 | 3 | 1 | 3 | 2 | 1 |
| 6. Rationale for choice of data collection tool/s | 3 | 3 | 3 | 3 | 3 | 1 | 3 | 1 | 3 |
| 7. The format and content of data collection tool is appropriate to address the stated research aim/s | 3 | 3 | 2 | 3 | 3 | 3 | 3 | 3 | 2 |
| 8. Description of data collection procedure | 1 | 2 | 3 | 3 | 3 | 2 | 3 | 3 | 1 |
| 9. Recruitment data provided | 2 | 3 | 3 | 3 | 1 | 1 | 3 | 3 | 2 |
| 10. Justification for analytic method selected | 3 | 0 | 2 | 3 | 1 | 0 | 0 | 3 | 2 |
| 11. The method of analysis was appropriate to answer the research aim/s | 3 | 1 | 3 | 3 | 2 | 3 | 3 | 2 | 2 |
| 12. Evidence that the research stakeholders have been considered in research design or conduct. | 0 | 3 | 3 | 3 | 1 | 1 | 3 | 3 | 3 |
| 13. Strengths and limitations critically discussed | 2 | 2 | 3 | 2 | 1 | 2 | 3 | 3 | 0 |

^(a)^For each criterion: minimum score 0, maximum score 3.

RCTs highlighted.

Table S1 continued. Quality assessment of included studies: all study designs (excluding case reports)

| **Quality Assessment with Diverse Studies (QuADS) appraisal criteria^(a)^** | Kenny  (2020) | Meeusen (2015) | Ojeda (2012) | Quintiliani (2018) | Schandrin  (2022) | Sharma  (2018) | Towsley  (2020) | Williams  (2013) |
| --- | --- | --- | --- | --- | --- | --- | --- | --- |
| 1. Theoretical or conceptual underpinning to the research | 3 | 2 | 1 | 2 | 3 | 1 | 3 | 3 |
| 2. Statement of research aim/s | 3 | 1 | 3 | 3 | 3 | 3 | 3 | 1 |
| 3. Clear description of research setting and target population | 3 | 1 | 3 | 1 | 3 | 3 | 3 | 2 |
| 4. The study design is appropriate to address the stated research aim/s | 3 | 2 | 2 | 2 | 3 | 3 | 3 | 1 |
| 5. Appropriate sampling to address the research aim/s | 3 | 0 | 2 | 1 | 3 | 3 | 3 | 1 |
| 6. Rationale for choice of data collection tool/s | 3 | 1 | 0 | 2 | 3 | 2 | 2 | 3 |
| 7. The format and content of data collection tool is appropriate to address the stated research aim/s | 3 | 2 | 2 | 1 | 2 | 2 | 2 | 1 |
| 8. Description of data collection procedure | 3 | 2 | 1 | 3 | 2 | 2 | 3 | 2 |
| 9. Recruitment data provided | 3 | 2 | 3 | 2 | 3 | 2 | 3 | 0 |
| 10. Justification for analytic method selected | 3 | 1 | 0 | 0 | 3 | 1 | 0 | 0 |
| 11. The method of analysis was appropriate to answer the research aim/s | 3 | 0 | 0 | 3 | 3 | 1 | 3 | 0 |
| 12. Evidence that the research stakeholders have been considered in research design or conduct. | 3 | 3 | 3 | 3 | 2 | 1 | 3 | 2 |
| 13. Strengths and limitations critically discussed | 3 | 1 | 0 | 2 | 3 | 3 | 2 | 1 |

^(a)^For each criterion: minimum score 0, maximum score 3

RCTs highlighted.

Table S2. Quality of reporting of included conference abstracts

| **Bespoke tool adapted from STROBE^(a)^** | Dash (2020) | Jayabalan (2014) | Naeem (2022) | Okuyama (2014) | Quintiliani (2020) |
| --- | --- | --- | --- | --- | --- |
| 1. Is the study design appropriate to address the stated aims & objectives? | No | Yes | No | Yes | Yes |
| 2. Is the primary outcome clearly defined? | Yes | Yes | Unclear | Yes | Yes |
| 3. Are data collection procedures clearly described? | Yes | Yes | No | Yes | Yes |
| 4. Are research setting(s) & target population(s) clearly described? | Yes | Yes | No | Yes | Yes |
| 5. Have methods of analysis been clearly described (including controlling for confounding, where appropriate)? | Unclear | No | No | No | Yes |
| 6. Are recruitment data reported? | Unclear | Unclear | No | Yes | No |
| 7. Are study results/findings clearly described? | Yes | Yes | Unclear | Yes | Yes |
| 8. Is there an interpretation of study results/findings? | Yes | Yes | Unclear | Yes | Yes |

^(a)^STROBE: Strengthening the Reporting of Observational Studies in Epidemiology [25]

RCTs highlighted.

Table S3. Quality assessment of included case reports

| **JBI^(a)^ Critical Appraisal Checklist for Case Reports** | Davin (2016) | Duker (2013) | Freund (2021) | Rocha (2013) | Singh (2015) |
| --- | --- | --- | --- | --- | --- |
| 1. Were patient’s demographic characteristics clearly described? | Yes | Unclear | Yes | Yes | Yes |
| 2. Was the patient’s history clearly described and presented as a timeline? | Yes | Yes | Yes | Yes | Yes |
| 3. Was the current clinical condition of the patient on presentation clearly described? | Yes | Yes | Yes | Yes | Yes |
| 4. Were diagnostic tests or assessment methods and the results clearly described? | Yes | Unclear | Yes | Yes | Yes |
| 5. Was the intervention(s) or treatment procedure(s) clearly described? | Yes | Yes | Unclear | Yes | Yes |
| 6. Was the post-intervention clinical condition clearly described? | Yes | N/A | N/A | Yes | Yes |
| 7. Were adverse events (harms) or unanticipated events (including ethicolegal issues) identified and described?^(b)^ | Unclear | Yes | Yes | No | No |
| 8. Does the case report provide takeaway lessons? | Yes | Yes | Yes | Yes | Yes |

^(a)^JBI: Joanna Briggs Institute [24]; ^(b)^We modified this criterion to include ethicolegal issues.
