# Supplementary material for: Video Recording Patients for Direct Care Purposes: Systematic Review and Narrative Synthesis of International Empirical Studies and UK Professional Guidance
Source: J Med Internet Res. 2023 Aug 16;25:e46478. doi: 10.2196/46478 (PMC10468707; doi:10.2196/46478)
Supplement: Multimedia Appendix 5 [file jmir_v25i1e46478_app5.docx]

**Multimedia Appendix 5**. Recruitment and retention rates as measures of video recording intervention acceptability.

Table S4. Recruitment and retention rates as measures of videorecording intervention acceptability

| Author  (Year) | Recruitment rate^(a)^ | Reasons for non-participation | Retention rate^(b)^ | Reasons for removal or withdrawal | Reasons associated with the video-recording intervention itself or other factors: TFA construct or ‘other’ category. |
| --- | --- | --- | --- | --- | --- |
| Amin  (2021) | 100% (60/60) | ^n/a^ | 90.0% (54/60) | Did not undergo study comparator (video-EEG) (n=6) | Other: study eligibility |
| Bayen (2017)^(d)^ | 66.7% (10/15) | _^(c)^ | 100% (10/10) | ^n/a^ | _^(c)^ |
| Bayen  (2021) | 34.2% (66/193) | N=27:  **- privacy concerns**  - family did not perceive a falls risk | 100%  (66/66) | ^n/a^ | **Intervention: ethicality**  Other: patient’s condition |
| Dash  (2016) | _^(c)^ | _^(c)^ | 91.8% (312/340) | *Video not captured* (n=28)*:*  - Duration or timing of seizures  **- Non-availability of caregiver and/or camera or phone**.  *Excluded after video capture (n=30):*  - Found to have psychogenic non-epileptic seizures. | **Intervention: self-efficacy**  Other: patient’s condition; study eligibility |
| Dash  (2020) | -^(c)^ | -^(c)^ | 73.4% (94/128) | *Video not captured (n=34):*  - Reasons not reported.  *Excluded after video capture (n=6):*  **- Video quality not suitable for clinical interpretation** | **Intervention: perceived effectiveness** |
| David  (2012) | -^(c)^ | -^(c)^ | 100%  (40/40) | ^n/a^ | ^n/a^ |
| DeVries  (2019) | -^(c)^ | -^(c)^ | 100%  (16/16) | ^n/a^ | ^n/a^ |
| Du Mortier  (2019) | 81.3%  (26/32) | _^(c)^ | 96.2%  (25/26) | No compulsions visible on video (n=1)  Declined to participate in study interviews (n=1). | *Unclear*  Other: study non-completion |
| Garfein  (2015) | 92.9%  (52/56) | **- confidentiality concerns (n=3)**  **- did not think he could keep the phone safe (n=1)** | 86.5%  (5/52) | **- Difficulties operating the smartphone (n=2)**  **- Preferred standard care (n=1)**  - Repeatedly failing to record videos (n=2)  - Drug susceptibility testing revealed drug-resistant TB after enrolment rendering participants ineligible (n=2) | **Intervention: ethicality; self-efficacy; affective attitude**  Other: study eligibility  *Unclear* |
| Guthrie  (2020) | 93.6%  (44/47) | - moderate physical disabilities and mental health conditions with heightened anxiety levels (n=3) | 95.4%  (42/44) | - Chose to delete their dysphagia videos – reasons not reported (n=2). | Other: patients’ condition  *Unclear* |
| Jayabalan  (2014) | _^(c)^ | _^(c)^ | _^(c)^ | _^(c)^ | _^(c)^ |
| Kenny  (2020) | 73.7%  (14/19) | **- apprehensive towards the tablet computer (n=4)**  - gave no reason (n=1) | 92.9%  (13/14) | - Discharged from hospital before trial commenced (n=1).  - Lost to follow up (n=2). | **Intervention: affective attitude**  Other: study non-completion |
| Meeusen  (2015) | 39.2%  (333/850)  survey response rate | _^(c)^ | N/A | *Reasons given by patients (n=34) for not watching the videorecording of their clinical encounter:*  **- not receiving a password to access the video online**  **- a problem understanding how to log in to the website**  **- video access had expired**  **- patient felt they did not need to watch the video**  **- bad internet connection affecting playability of the video.** | **Intervention: other – technical; intervention coherence; affective attitude** |
| Naeem  (2022) | _^(c)^ | _^(c)^ | _^(c)^ | _^(c)^ | _^(c)^ |
| Ojeda  (2012) | 49.4%  (132/267) | n=135  - Low seizure frequency  - Short duration of seizures  **- Non-availability of caregiver** | 16.7%  (22/132) | n=110:  - Not witnessing any seizure  - Short duration of seizure  - Nocturnal seizures  - Emotional blackout | **Intervention: self-efficacy**  Other: patient’s condition |
| Okuyama  (2014) | 76.5% (13/17) | _^(c)^ | 69.2%  (9/13) | _^(c)^ | _^(c)^ |
| Quintiliani  (2018) | _^(c)^ | _^(c)^ | 93.8%  (15/16) | - Thought that making a video declaration about end-of-life decisions would be too emotionally draining (n=1). | **Intervention: burden** |
| Quintiliani  (2020) | _^(c)^ | _^(c)^ | _^(c)^ | _^(c)^ | _^(c)^ |
| Schandrin  (2022) | 50.4%  (60/119) | **- declined to be filmed (n=23)**  - lost to follow up before inclusion (n=17)  **- declined to participate in the study (n=16)**  - other reasons (not reported) (n=3) | 43.3%  (26/60) | **- withdrew (n=11)**  - no-shows in follow up (n=22)  - unable to proceed, non-compatible state (n=1) | _^(c)^ |
| Sharma  (2018) | _^(c)^ | _^(c)^ | 39.6%  (40/101) | _^(c)^ | _^(c)^ |
| Towsley  (2020) | 60.6%  (20/33) | **- not interested (n=4)**  - not at this time (n=3)  - gave other reasons (n=6) including: “too old”, did not want to sign consent, **did not want to be video recorded, or felt self-conscious about making a video (e.g. missing teeth, impaired speech).** | 90.0%  (18/20) | - withdrawn at family’s request: no reason given (n=1)  - discharged (n=1) | **Intervention: affective attitude**  Other: study non-completion |

^(a)^Proportion of eligible patients recruited; ^(b)^Proportion of patient participants included in analyses assessing the study’s primary outcome. ^(c)^Recruitment rate and/or retention rate and/or reasons for non-participation, removal or withdrawal not reported by study authors; rates cannot be calculated from reported data. n/a: not applicable. Williams (2012) report a feasibility study with one patient-carer dyad only; case reports also excluded from this table.
