# Supplementary material for: Video Recording Patients for Direct Care Purposes: Systematic Review and Narrative Synthesis of International Empirical Studies and UK Professional Guidance
Source: J Med Internet Res. 2023 Aug 16;25:e46478. doi: 10.2196/46478 (PMC10468707; doi:10.2196/46478)
Supplement: Multimedia Appendix 6 [file jmir_v25i1e46478_app6.docx]

**Multimedia Appendix 6.** Acceptability and perceived effectiveness of video recording patients for direct care purposes: subthemes and additional verbatim quotes.

Table S5: Acceptability of video-recording patients for direct care purposes: sub-themes and verbatim quotes

| **TFA Construct** | **Sub-themes^(a)^** | **Additional sub-themes** | **Extracts from study findings/ verbatim quotes from study participants** |
| --- | --- | --- | --- |
| **Affective Attitude** | Positively engaged  [28,30,45] | - | *- ‘Most service-users (88% n=37) showed interest and were keen to view themselves’* (Extract; Guthrie 2020). |
|  | Eager to share  [30,45] | - | *- ‘The vast majority of patients were excited about the prospect of being recorded and ability to share the videos with friends and family members and their other physicians.’* (Extract; Sharma 2018) |
|  | Helpful  [36-37,46,47] | *-* | *‘Overall, 263 patients (88.0%) indicated the video was helpful to them in some way, and 259 of these respondents (98.5%) indicated they would also recommend the video service to other patients.’* (Extract; Meeusen 2015) |
|  | Reassured  [28,37] | *-* | *“I felt more reassured that they were having a visual representation of how the movement should be being produced, which reassured me a little bit more than just giving the verbal, ‘’this is what you need to be doing and remember that and do that.”* (Participant-Physiotherapist; Kenny 2022) |
|  | True representation  [28,36,38] | *-* | *‘In the post video viewing surveys, family and staff, generally, rated the video positively […] as accurate, adequate, complete, and credible.’* (Extract; Towsley 2020) |
|  | Would recommend  [35-37,47] | *-* | *‘8 [participants] (89%) agreed/strongly would probably or definitely recommend this to other patients.’* (Extract; Okuyama 2014) |
| **Burden** | Easy process  [45,32,34,38,39] | Use of mobile device | *‘the mobile device app provided to review videos proved to be accessible and easy to use to facility staff’* (Extract; Bayen 2017) |
|  |  | No added burden | *‘Overall, 92% reported never/rarely having problems recording videos.*’ (Extract; Garfein 2015) |
|  | Additional burden  [28,37,39,47] | Technical issues | *‘We did not anticipate the number and complexity of technological and data security requirement challenges that were encountered in operationalizing the project’* (Extract, Williams 2013) |
|  |  | Device security | *“We were always like on edge all the time making sure we knew where the iPads were, making sure it’s locked up”* (Participant-Physiotherapist; Kenny 2022) |
|  |  | Worried about appearance | *‘Those who thought the process was less easy indicated that they wished their appearance in the video was better’* (Extract, Quintiliani 2018) |
|  |  | Need time to prepare | *‘Those who thought the process was less easy indicated that […] they needed time to think and plan what to say’* (Extract, Quintiliani 2018) |
| **Ethicality** | Sense of control  [48] | - | *‘The large majority of patients (81%) indicated that it would be important to have a sense of control over the camera […] “Actually, it’s crucial to be able to turn the camera on and off whenever I feel like having a private moment.” (patient 16). 50% of patients said they would like to see the recordings (and maybe even delete footage) before it would be shared with researchers or medical personnel.’* (Extract & verbatim quote; De Vries 2019) |
|  | Consent of others  [48] |  | *“Other people should be kept out of the video as much as possible... but this is probably not possible, so my partner needs to consent as well” (patient 7).* (Participant; De Vries 2019) |
| **Intervention coherence** | Understanding how the intervention works  [28,45,33] |  | *‘Most participants (92%, n= 22) elaborated on how they gained a greater insight into their symptoms and became more motivated to change by watching the video. They mentioned three themes:(a) ‘Being an observer’ rather than a participant, leading to more insight and awareness (92%, n= 22). (b) Insight and awareness made it difficult to ignore the serious-ness of their compulsions (58%, n= 14). (c) Seeing progress on the video shows that treatment works (25%, n= 6)* (Extract; Du Mortier 2019) |
|  | Unfamiliar with technology  [45] |  | *‘Some service-users showed curiosity about the iPad and appeared not to recognise or understand it. One person asked if it was a mirror and looked to brush her hair, another asked if it could continue to see him in another room.’* (Extract; Guthrie 2020) |
|  | Optimising the intervention  [28] |  | *‘Therapists proposed a number of changes to the user interface […] using older videos to demonstrate progress, and goal attainment […] using the tablet to monitor the amount of independent exercise undertaken.’* (Extract; Kenny 2022) |
| **Opportunity costs** | Privacy  [48] |  | *- “I don’t want a camera in every room at the same time, I need to have some privacy somewhere.” (patient 8)* (Participant; De Vries 2019) |
|  | No detrimental impact  [30,32] |  | *- ‘The incorporation of the video recordings into our clinic flow did not add any significant time to the visit’* (Extract; Sharma 2018)  *- ‘In the present study, no patient made a comment that the video was intrusive or that they felt the video had made them feel uneasy’* (Extract; Meeusen 2015) |
| **Self efficacy** | Cognitive impairment  [28] |  | *‘Cognitively impaired patients were less likely to engage with the tablet: “…other than when (the physiotherapy assistant) went in or we incorporated it as part of a session”’* (Extract & participant quote; Kenny 2022) |
|  | Computer literacy  [47] |  | *‘Ten other respondents experienced a computer literacy problem, where they indicated it was difficult for them to figure out how to get to the video, even without any technical problems being present.’* (Extract; Meeusen 2015) |
|  | Psychological state  [37,41] |  | *‘In terms of re-commending this process to others, one patient who gave a rating of ‘neutral’ indicated that the video declaration process might not be appropriate for certain people depending on their illness or psychological state’* (Extract; Quintiliani 2018) |
| ***Other*** | **Operational pressures**  [32] |  | *‘Facility management reported hardly ever using the video feeds during [the first 7 weeks] because of the numerous other challenges faced with operating a memory care facility and the little obvious value granted to the video so far.’* (Extract; Bayen 2017) |
| ***Other:*** | **Environment**  [28,38] |  | *‘One resident mentioned technical issues during the [video-recording] such as noise from outside.’* (Extract; Towsley 2020) |

^(a)^Papers containing study data linked to a theme are cited in brackets.

Table S6: Perceived effectiveness: sub-themes and verbatim quotes

| **Main Themes** | **Sub-themes** | **Verbatim quotes from study participants/ Extracts from study findings** |
| --- | --- | --- |
| Supports patient assessment  [32-34,39,45] | Assess condition severity | - ‘useful to grade the severity of the injury’ (Extract; Bayen 2017)  - ‘These aspects of SLT assessment are difficult to measure and record accurately in a format that allows subsequent comparison over time for quality and severity’ (Extract; Guthrie 2020)  *- “I think the use of iPad has a huge advantage, you get a true visual representation which helps to identify specific concerns, progress, improvements/decline.”* (Participant – Speech & Language Therapist; Guthrie 2020)  - *“Visiting community staff will be able to view this footage as part of their assessments [for resettlement planning], due to difficulties coordinating a visit when the service-user is likely to have a meal….”* (Participant – Speech & Language Therapist; Guthrie 2020). |
|  | Identify change or improvement |  |
|  | True visual representation |  |
|  | Review any time |  |
|  | Enable remote assessment |  |
| Supports care or treatment  [28,29,32,33,47] | Informs preventative care measures | - ‘facility management carried out preventative care interventions, which they believed would address some of the causes of future falls’ (Extract, Bayen 2017)  - *“I watched the video. I thought, ‘I could do them [the exercises] at first’ . . . when I watched the video again, I thought, ‘well I haven’t been doing that right’. When I watched you do it, I know I have to do it properly”* (Participant-patient with stroke; Kenny 2022)  - *“Then I think “goodness, that’s a bit much”. There I go, wiping behind the faucet with the cloth again. I can’t help thinking, “hang on I’ve done that ten times already”. Do I really do it so many times? That’s excessive.”* (Participant-patient with obsessive compulsive disorder; Du Mortier 2019) |
|  | Facilitates rehabilitation |  |
|  | Enhances patient insight |  |
|  | Reduces patient anxiety |  |
| Promotes patient engagement & involvement  [28,33,34,45] | In assessment | - ‘The use of video gave immediate visual information to support the verbal discussion enhancing the participation of service-users who had limited communication and attention, enabling involvement with the assessment process: *“Just seeing that I realise how bad I am getting now”*’ (Service-user with dysphagia; Guthrie 2020)  - “the [videorecording on my] phone was very convenient [for me]. I could take my pills on my own time instead of waiting for someone to watch me.” (Patient participating in a medication adherence intervention for tuberculosis; Garfein 2015) |
|  | In care or treatment |  |
| Improves communication patients, care-givers, & professionals  [38,39,45,47,48] | Communicate problems or symptoms | - *“When you visit a neurologist you really need to describe the problem the right way and you never know exactly how often symptoms appear.”* (Neurology Patient; de Vries 2019)  - ‘Some residents stated that by creating and sharing the video, they felt better that certain preferences had been conveyed including their desire for family involvement, care near EOL [end of life], and feelings about dying.’ (Extract; Towsley 2020)  - ‘video being better than expressing wishes through phone calls or in text’. (Extract; Quintiliani 2018) |
|  | Communicate care preferences |  |
|  | Support patient-careprovider discussion |  |
|  | Support multidisciplinary communication | *- “Visiting community staff will be able to view this footage as part of their assessments.”* (Participant-Professional; Guthrie 2020)  - ‘The video footage offered opportunities for the SLT to seek support and second opinions from supervising SLTs.’ (Extract; Guthrie 2020) |
|  | Obtain advice or second opinion |  |
| Improves recall of clinical information  [28,45,47] | Relating to the patient’s condition | *- “Very helpful clinically, reduces reliance upon our memory and written assessment over long periods of time.”* (Psychiatrist; Guthrie 2020)  *- “Each time I re-watch the video I discover additional things. No way I could remember all this critical information without this video."* (Neurosurgical patient; Meeusen 2015) |
|  | Relating to the clinical consultation |  |
| No benefit  [38,47] | | - ‘About half of the residents conveyed they had not noticed any benefits to sharing their video, and most did not identify ways the video had been helpful in their care.’ (Extract; Towsley 2020)  *- “Really, the video was not needed. My situation was not complicated at all, so there was nothing mentioned that was difficult to remember. However, the video would be extremely helpful if the situation were more complicated.”* (Neurosurgical patient; Meeusen 2015) |

TFA: Theoretical Framework of Acceptability [21]
